# Supplementary material for: ERBB2 signaling drives immune cell evasion and resistance against immunotherapy in small cell lung cancer
Source: Nat Commun. 2025 Dec 9;16:10983. doi: 10.1038/s41467-025-66800-x (PMC12689756; doi:10.1038/s41467-025-66800-x)
Supplement: Supplementary file 1 — Supplementary Information [file 41467_2025_66800_MOESM1_ESM.pdf]

Supplementary Information

ERBB2 signaling drives immune cell evasion and resistance against immunotherapy in small cell lung cancer

Supplementary Figures 1-11

Supplementary Fig. 1: MHC-I KO in SCLC cells associates with reduced infiltration of CD8 positive T cells.
Supplementary Fig. 2: ERBB2 in metastatic SCLC is not driven by canonical oncogenic pathways or genomic alterations.
Supplementary Fig. 3: ERBB2 inhibition/KO silences AKT and MAPK signaling and restores antigen presentation programs.
Supplementary Fig. 4: ERBB2 KO and WT show similar *in vivo* growth in immunodeficient mouse model.
Supplementary Fig. 5: ERBB2i leads to a STING dependent MHC-I upregulation and leads to prolonged OS with ERBB2 inhibitor lapatinib.
Supplementary Fig. 6: ERBB2 KO in SCLC cells leads to prolonged survival *in vivo* compared to SCLC wild type.
Supplementary Fig. 7: T cell depletion abrogates immune control of ERBB2 deficient SCLC tumors.
Supplementary Fig. 8: ERBB2 inhibition does not affect neuroendocrine differentiation.
Supplementary Fig. 9: ERBB2 regulates MHC-I via MAPK-AKT-TBK1 signaling.
Supplementary Fig. 10: High ERBB2 expression is associated with a shorter overall survival in the immunochemotherapy patient cohort.
Supplementary Fig. 11: ERBB2 inhibition combined with anti-PD-1 blockade enhances MHC-II expression and reduces ERBB2 expression in mice suffering from SCLC.

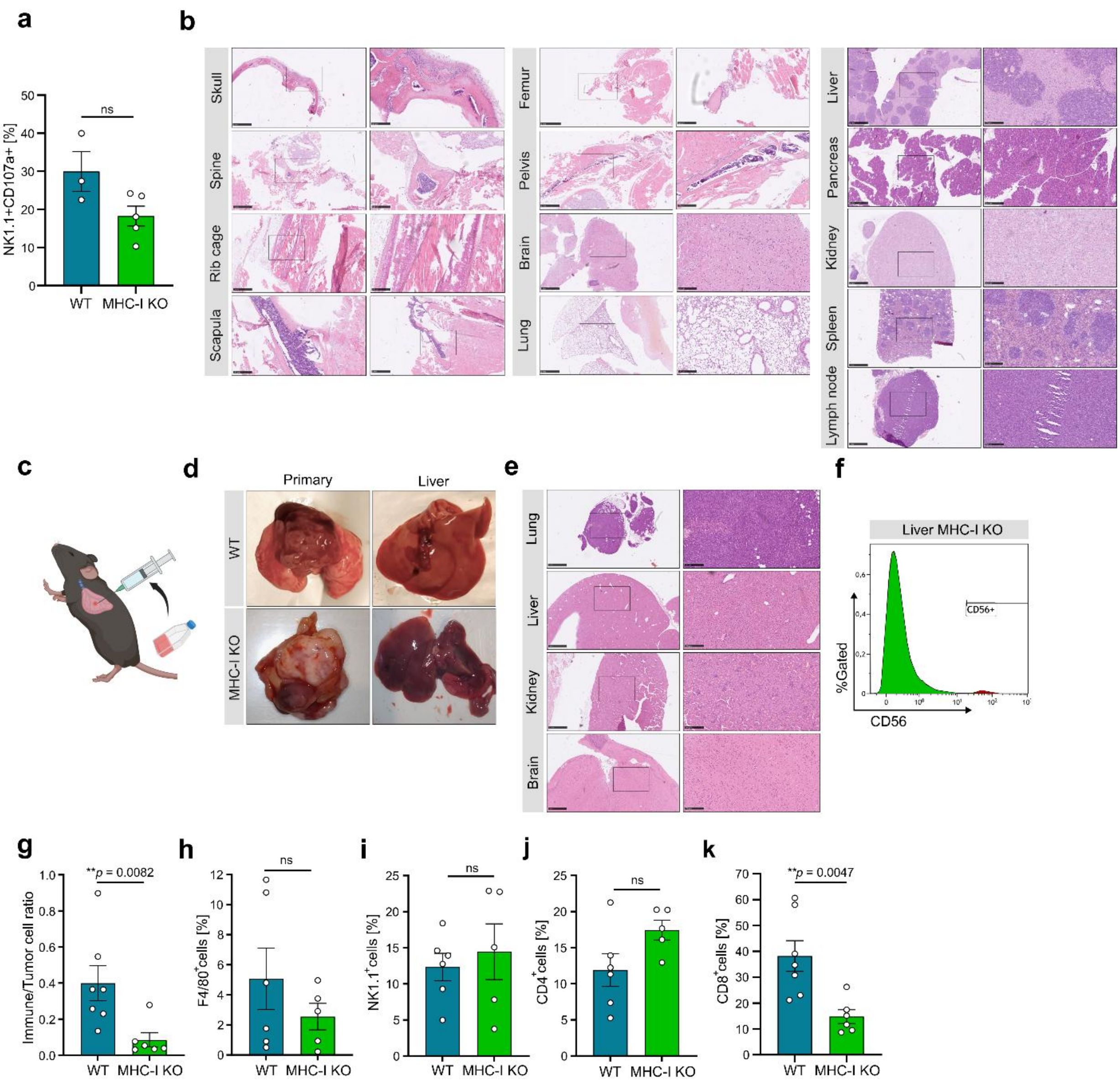

Supplementary Fig. 1: MHC-I KO in SCLC cells associates with reduced infiltration of CD8 positive T cells. a, Activated NK cells in liver of MHC-I KO i.v. injected BL6 mice in percent measured by flow cytometry (n = 3 and 5, respectively). **b,** Representative potential metastatic regions of MHC-I KO i.v.-injected mice with metastatic settlement in liver and lymph node. Scale bars: 1 mm (overview, left), 250 µm (right). **c,** Illustration of orthotopic injection of MHC-I KO cells into immunocompetent BL6 mice. Created in BioRender. Meder, L. (2025) https://BioRender.com/a7q733t. **d,** Representative images of harvested lungs and respective liver after sacrifice of WT (n = 7) or MHC-I-KO (n = 6) injected mice. **e,** Representative H&E-stained sections of lung, liver, kidney and brain from WT and MHC-I KO orthotopically injected mice. Scale bars, 1 mm (overview, left), 250 µm (right). **f,** Representative flow cytometry histogram showing CD56 expression in harvested liver tissue from mice after orthotopic MHC-I KO injection. **g-k** Immune cell infiltration in the lungs from WT and MHC-I KO orthotopically injected mice determined by flow cytometry. **g,** Ratio of immune and tumor cells determined via the relative expression of CD45 and CD56-positive cells (n = 7 and 6, respectively). **h,** F4/80 expression (n = 6 and 5, respectively). **i,** NK1.1 expression (n = 6 and 5, respectively). **j,** CD4 expression (n = 6 and 5, respectively). **k,** CD8 expression (n = 7 and 6, respectively). Statistical analysis for all comparisons was performed using two-sided Mann-Whitney test. Data are presented as mean ± SEM. ns, not significant, $**p < 0.01$. Source data are provided as a Source Data file.

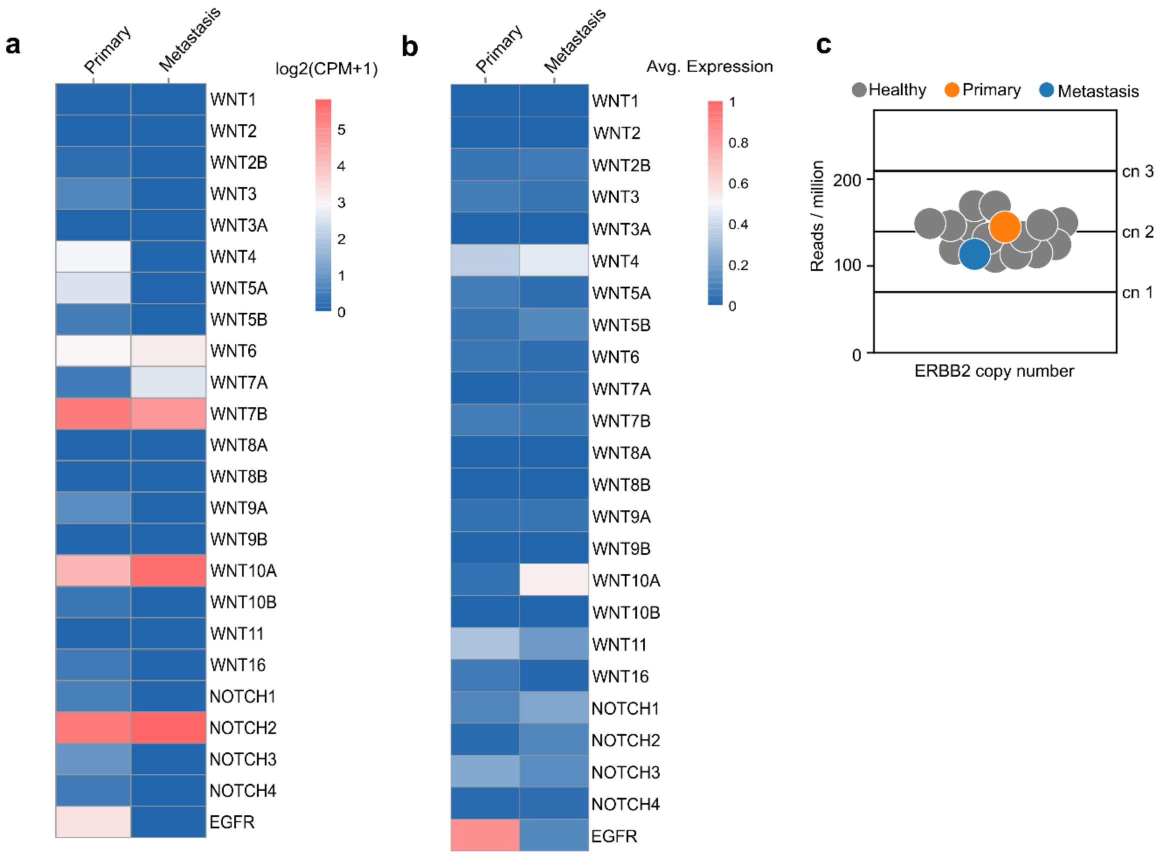

Supplementary Fig. 2: ERBB2 in metastatic SCLC is not driven by canonical oncogenic pathways or genomic alterations. a, RNA-seq analysis comparing combined primary murine SCLC cell lines (n = 3, biological replicates) to a liver metastasis derived murine SCLC cell line. **b,** scRNA-seq analysis of primary and metastatic SCLC tumors from 19 patients (primary n = 9, metastasis n = 12). **c,** Copy-number variation analysis comparing primary and liver metastatic murine SCLC cell line (n = 1). Source data are provided as a Source Data file.

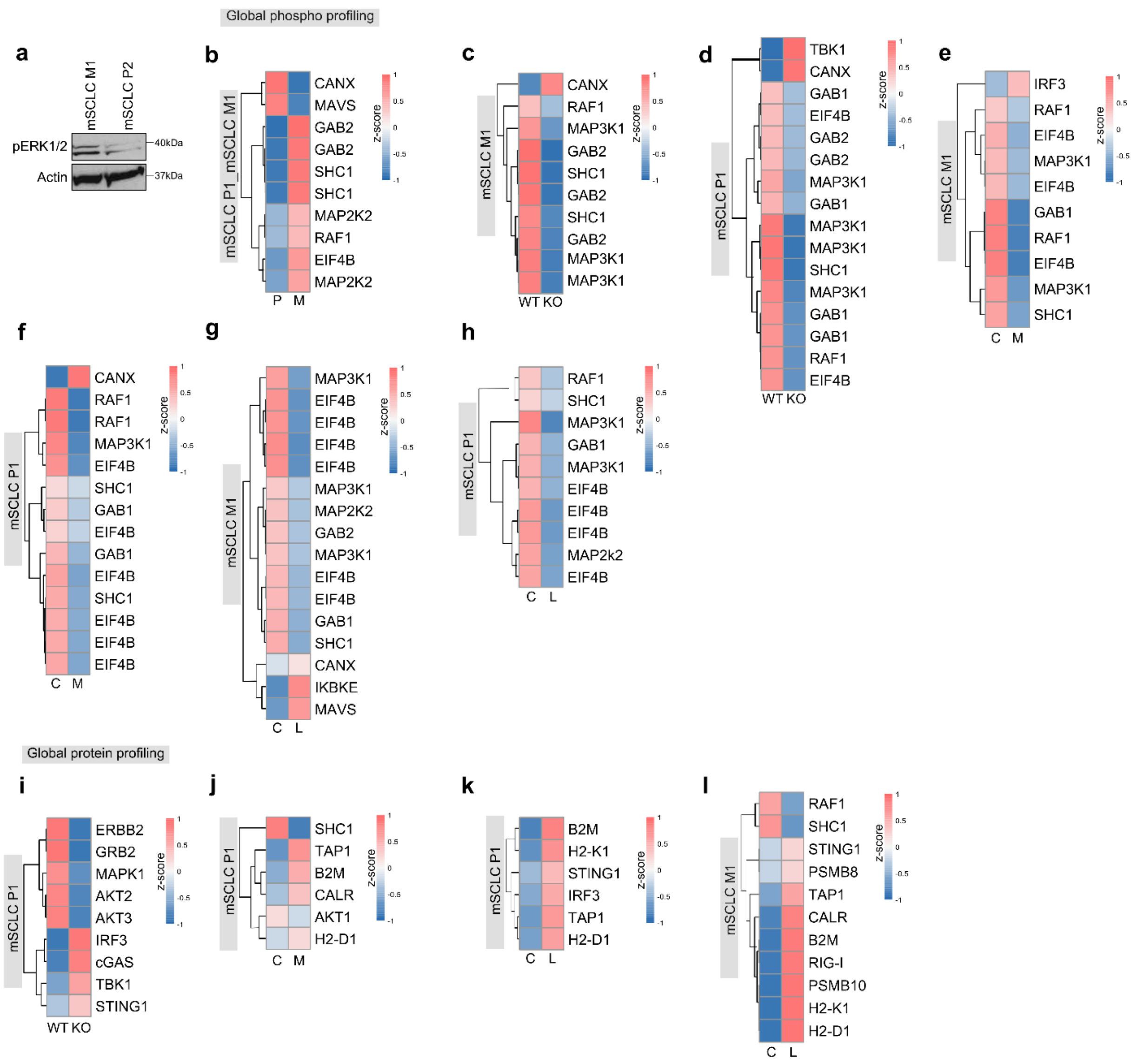

Supplementary Fig. 3: ERBB2 inhibition/KO silences AKT and MAPK signaling and restores antigen presentation programs. a, ERK phosphorylation in primary and metastatic murine SCLC cell lines determined by Western blot. **b–h,** Heatmaps showing differentially phosphorylated proteins in global phospho-profiling across conditions in murine SCLC cell lines (z-score normalized, biological replicates n = 3 per group). **b,** Primary vs. metastatic SCLC cell line. **c,** WT vs. ERBB2 KO in metastatic SCLC cell line. **d,** WT vs. ERBB2 KO in metastatic SCLC cell line. **e,** Control (untreated) vs. 24 h mubritinib-treated metastatic SCLC cell line. **f,** Control (untreated) vs. 24 h mubritinib treated primary SCLC cell line. **g,** Control (untreated) vs. 24 h lapatinib treated metastatic SCLC cell line. **h,** Control (untreated) vs. 24 h lapatinib treated primary SCLC cell line. **i-j,** Heatmaps showing differentially expressed proteins in global protein profiling across conditions in murine SCLC cell lines (z-score normalized, biological replicates n = 3 per group). **i,** WT vs. ERBB2 KO in primary SCLC cell line. **j,** Control (untreated) vs. 24 h mubritinib (1 µM) treated primary SCLC cell line. **k,** Control (untreated) vs. 24 h lapatinib treated primary SCLC cell line. **l,** Control (untreated) vs. 24 h lapatinib treated metastatic SCLC cell line. ERBB2 inhibitors were used at a concentration of 1 µM. Source data are provided as a Source Data file.

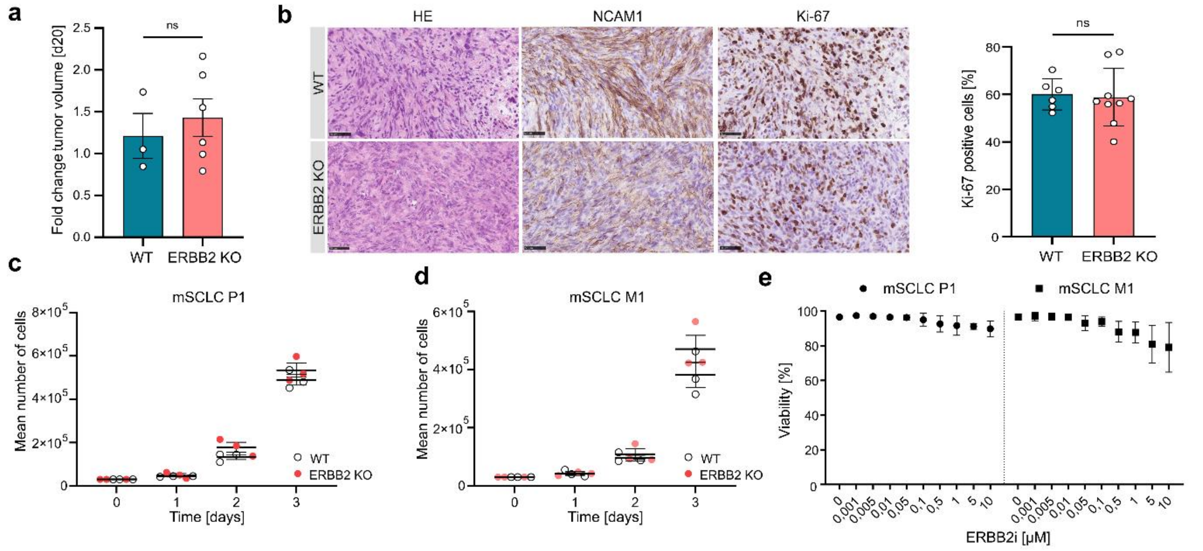

Supplementary Fig. 4: ERBB2 KO and WT show similar *in vivo* growth in immunodeficient mouse model. a, Fold change of tumor volume in ERBB2 KO or WT subcutaneously injected NSG mice at day 20 (two-sided Mann-Whitney test; n = 3 and 6, respectively). **b,** Representative images of H&E, NCAM1 and Ki-67 IHC stain of SCLC WT and ERBB2 KO subcutaneously injected immunodeficient NSG mice. Scale bars: 50 µm. Corresponding quantification of Ki-67 (two-sided Mann-Whitney test; n = 3 ROIs/animal) **c,** Proliferation of WT vs. ERBB2 KO of murine SCLC primary cell line (two-way ANOVA; n = 1 per group). **d,** Proliferation of WT vs. ERBB2 KO of murine SCLC metastatic cell line (two-way ANOVA; n = 1 per group). **e,** Flow cytometric analysis of cell death in primary (left) and metastatic (right) murine SCLC cell lines. Cells were untreated or treated with increasing concentrations of mubritinib for 48 h, before analysis of apoptosis and necrosis by simultaneous detection of cell-surface annexin V and DNA binding of propidium iodide (n = 3 biological replicates). Data are presented as mean ± SEM. ns, not significant. Source data are provided as a Source Data file.

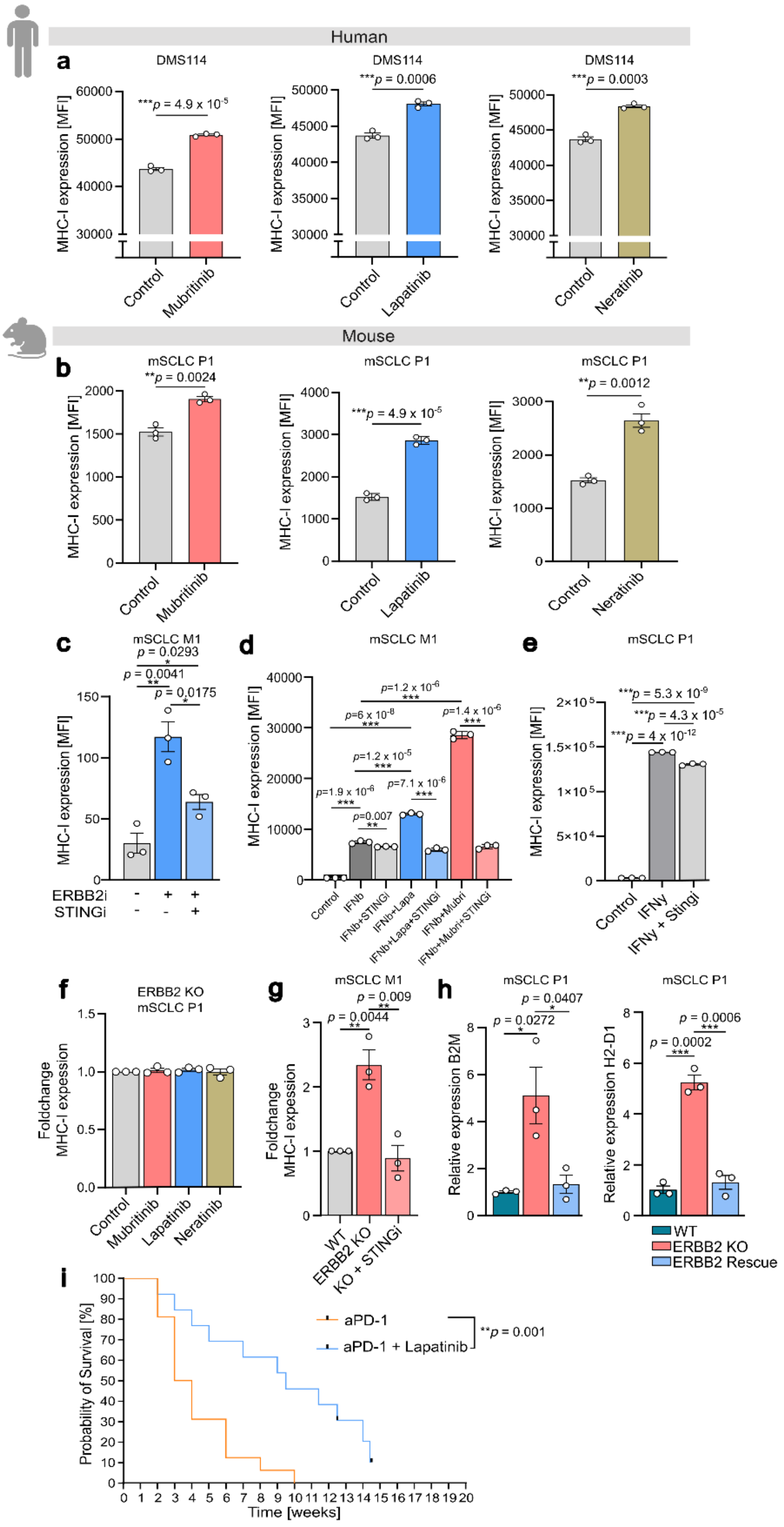

Supplementary Fig. 5: ERBB2i leads to a STING-dependent MHC-I upregulation and leads to prolonged OS with ERBB2 inhibitor lapatinib. a, MHC-I expression determined by flow cytometry in human SCLC cell line DMS114 untreated or treated with mubritinib, lapatinib and neratinib (1 µM each) (two-sided, unpaired Student's t-test; n = 3 biological replicates). **b,** MHC-I expression determined by flow cytometry in murine SCLC primary cell line untreated or treated with mubritinib, lapatinib and neratinib (1 µM each) (two-sided, unpaired Student's t-test, n = 3 biological replicates). **c,** MHC-I expression determined by flow cytometry of murine SCLC metastatic cell line untreated or treated with ERBB2 inhibitor lapatinib alone or in combination with STING inhibitor (1 µM each) (two-sided,

unpaired Student's t-test; n = 3 biological replicates). **d,** MHC-I expression quantified by flow cytometry in murine SCLC metastatic cells untreated or treated with IFNβ (10 ng/ml) alone or in combination with STING inhibitor (1 μM), lapatinib, or mubritinib, as indicated (two-sided, unpaired Student's t-test; n = 3 biological replicates). **e,** MHC-I expression measured by flow cytometry in murine SCLC cell primary line treated with IFNγ (40 ng/ml) alone or in combination with a STING inhibitor (1 μM) (two-sided, unpaired Student's t-test; n = 3 biological replicates). **f,** Foldchange in MHC-I expression determined by flow cytometry in ERBB2 KO untreated or treated with mubritinib, lapatinib and neratinib (1 μM each) (n = 3 biological replicates). **g,** WT vs. ERBB2 KO in murine SCLC cell line without and with STING inhibition (1 μM) (two-sided, unpaired Student's t-test; n = 3 biological replicates). **h,** Quantitative RT-PCR analysis of *B2M*, *H2-D1* expression in ERBB2 WT, ERBB2 KO and ERBB2 rescued SCLC cells (two-sided, unpaired Student's t-test, n = 3 biological replicates). **i,** Overall survival of *Rb1*/*Trp53*-depleted SCLC tumor mice treated with anti–PD-1 monotherapy (aPD-1, orange, n = 16) or with combination therapy with anti–PD-1 and ERBB2 inhibitor lapatinib (aPD-1 + lapatinib, blue, n = 13) (log-rank (Mantel-Cox test)). a-h are shown as mean ± SEM. $*p < 0.05$, $**p < 0.01$, $***p < 0.001$. Source data are provided as a Source Data file. Icons created in BioRender. Meder, L. (2025) https://BioRender.com/z2ehzfh.

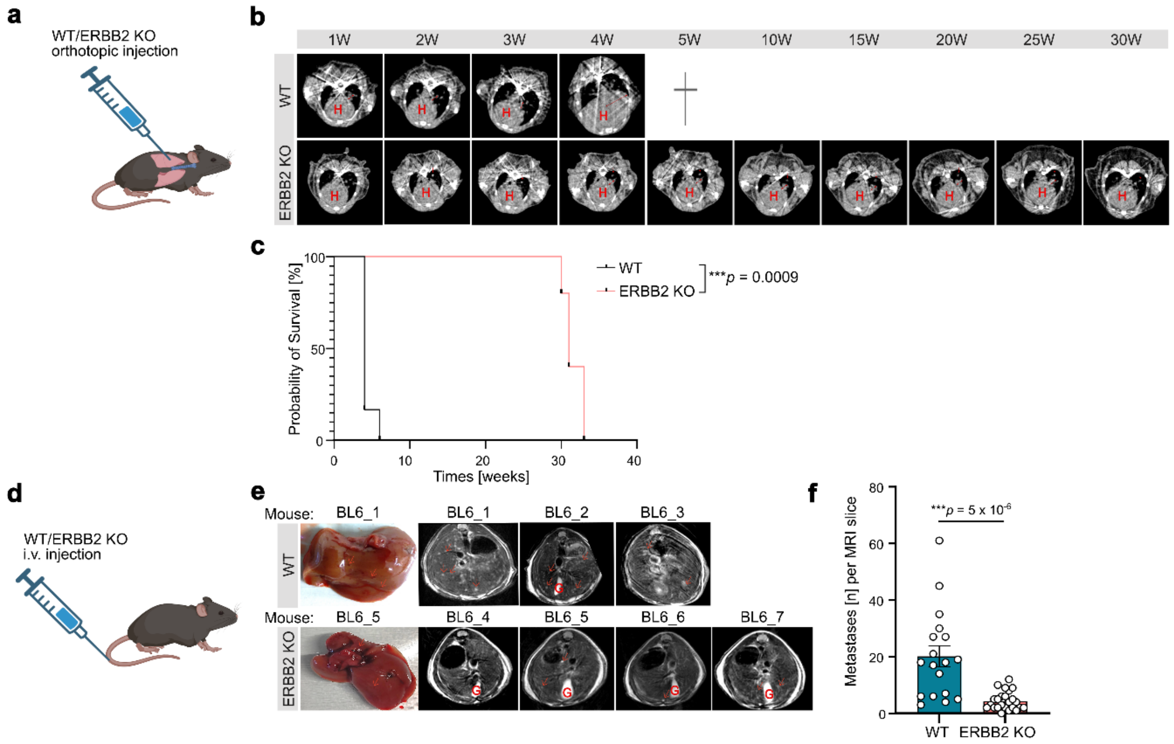

Supplementary Fig. 6: ERBB2 KO in SCLC cells leads to prolonged survival *in vivo* compared to SCLC wild type. a, Schematic representation of orthotopic injection of mSCLC M1 WT or ERBB2 KO in immunocompetent BL6 mice. Created in BioRender. Meder, L. (2025) https://BioRender.com/y4lktuu. **b,** Serial µCT measurements of one representative mouse per therapy group. Target lesion diameter is marked red. H, heart; cross (hand-drawn), dead. **c,** Overall survival of orthotopic ERBB2 KO and WT injected mice, (log-rank (Mantel–Cox test); WT, n = 6; ERBB2 KO, n = 5). **d,** Schematic representation of i.v. injection of mSCLC P4 WT or ERBB2 KO in immunocompetent BL6 mice. Created in BioRender. Meder, L. (2025) https://BioRender.com/y4lktuu. **e,** MRI measurements of one representative mouse per therapy group, 3 weeks post injection. Target lesion diameter is marked in red. **f,** Quantification of metastasis per MRI slice (two-sided Mann-Whitney test; WT n = 3 mice, ERBB2 KO n = 4 mice; 5 MRI slices per mouse; error bars mean ± SEM). ***p < 0.001, Source data are provided as a Source Data file.

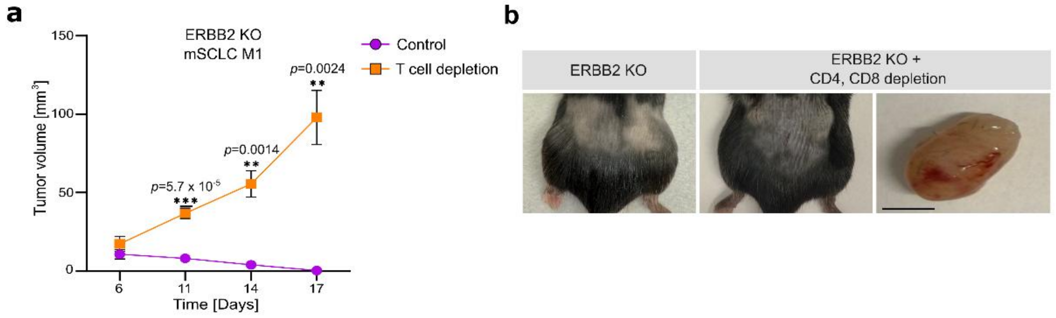

Supplementary Fig. 7: T cell depletion abrogates immune control of ERBB2 deficient SCLC tumors. a, Tumor volume of subcutaneously injected mice with ERBB2 KO with and without T cell depletion (multiple two-sided, unpaired Student's t test with Bonferroni correction, n = 6 per group). **b,** Representative images of subcutaneously ERBB2 KO-injected mice and respective tumor with or without T cell depletion (n = 6 per group). Scale bar: 5 mm. Data are shown as mean ± SEM. ***p* < 0.01, ****p* < 0.001. Source data are provided as a Source Data file.

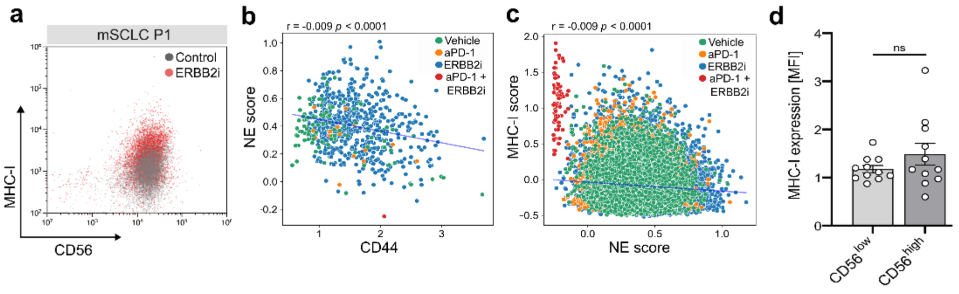

Supplementary Fig. 8: ERBB2 inhibition does not affect neuroendocrine differentiation. a, Representative scatter plot of MHC-I and CD56 expression after treatment of murine SCLC cell line with ERBB2i (lapatinib, 1 µM) for 24 h. **b,** Scatter plot of neuroendocrine (NE) score versus CD44 expression based on scRNA-seq of lung tumor cells from the four treatment groups vehicle, anti–PD-1, ERBB2i (mubritinib) and combination therapy (mubritinib + anti–PD-1) (Pearson correlation analysis; n = 4). **c,** Scatter plot of MHC-I versus neuroendocrine (NE) score based on scRNA-seq of lung tumor cells from the four treatment groups (Pearson correlation analysis; n = 4). **d,** MHC-I expression in CD56low, CD56high tumor cells measured by flow cytometry in autochthonous SCLC mice (two-sided Student's t-test; ns, not significant; error bars, mean ± SEM; vehicle n = 9, ERBB2i mubritinib n = 2). ns, not significant. Source data are provided as a Source Data file.

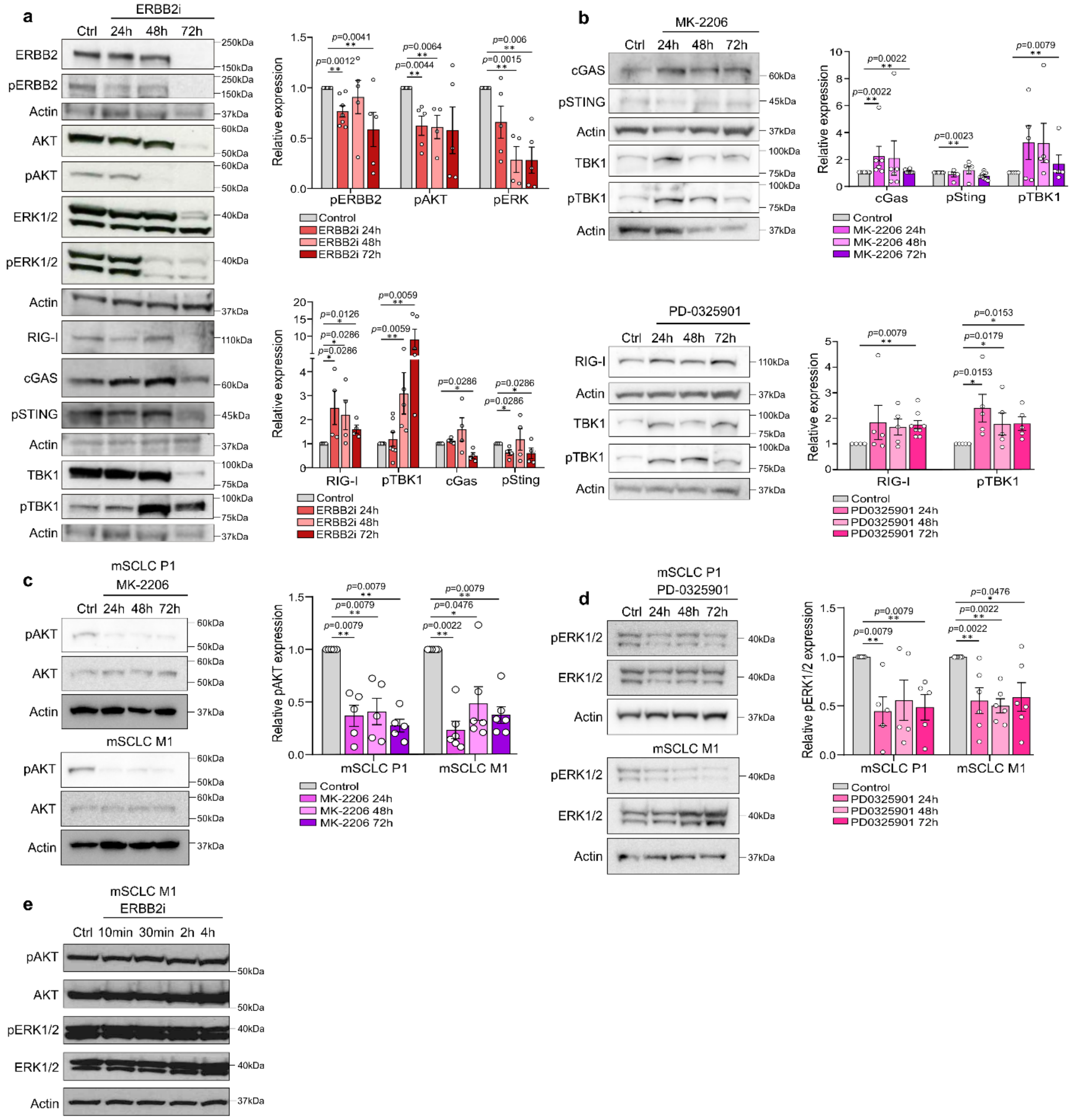

Supplementary Fig. 9: ERBB2 regulates MHC-I via MAPK-AKT-TBK1 signaling. a, Western blot and corresponding quantification of protein relative quantity or phospho-protein status in mSCLC M1 treated with DMSO (1 µL, control) or ERBB2i (1 µM) for 24 to 72 hours (two-sided Mann–Whitney test; n = 4 and 5, respectively). **b,** Western blots and corresponding quantification of mSCLC M1 treated with DMSO (1 µL, control), MK-2206 (1 µM) or PD-0325901 (1 µM) for 24 to 72 hours (n = 4 and 5, respectively). **c,** Western blots and corresponding quantification of mSCLC P1 and mSCLC M1 treated with DMSO (1 µL, control) or MK-2206 (1 µM) for 24 to 72 hours (two-sided Mann–Whitney test; n = 4 and 5, respectively). **d,** Western blots and corresponding quantification in mSCLCP1 and mSCLCM1 treated with DMSO (1 µL, control) or PD-0325901 (1 µM) for 24 to 72 hours (two-sided Mann–Whitney test; mSCLC P1 n = 4 and 5, respectively). **e,** Western blots of mSCLC M1 treated with ERBB2 inhibitor mubritinib (1 µM) for 10 min, 30 min, 2 h and 4 h. Western blots were quantified by densitometry, and band intensities were normalized to β-Actin and expressed relative to the control group. Data are represented as mean ± SEM. $*p < 0.05$, $**p < 0.01$. Source data are provided as a Source Data file.

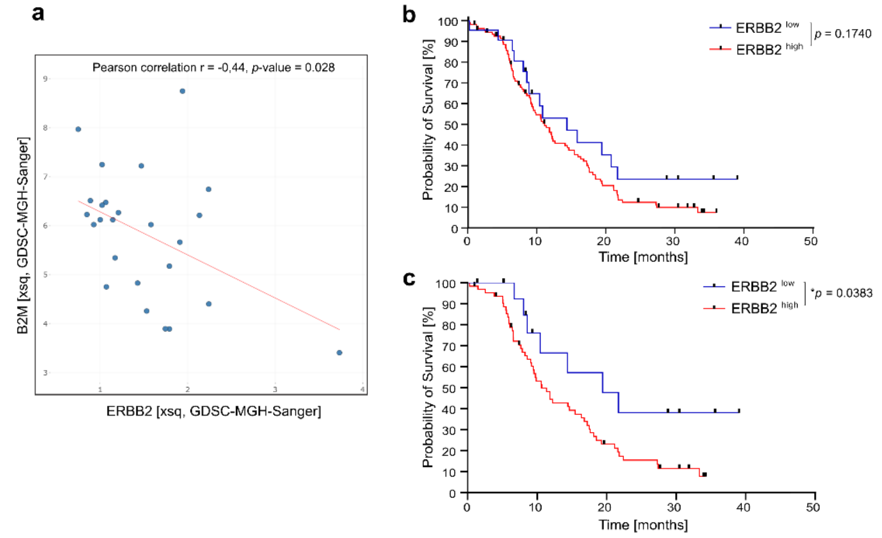

Supplementary Fig. 10: High ERBB2 expression is associated with a shorter overall survival in the immunochemotherapy patient cohort. a, RNA expression data from human SCLC cell lines from GDSC-MGH-Sanger provided by CellMinerCDB within the SCLC dataset. **b,** Overall survival of ERBB2 low vs. high patients in the carboplatin, etoposide + atezolizumab treatment arm of IMpower133 (n = 132). **c,** Overall survival in the TMB-high subgroup of ERBB2 low vs. high patients in the IMpower133 trial cohort (n = 78). Expression cutoff was determined using the Cutoff Finder[42]. Statistical analysis was done using the Mantel-Cox (log-rank) test (*, *p* < 0.05). Source data are provided as a Source Data file.

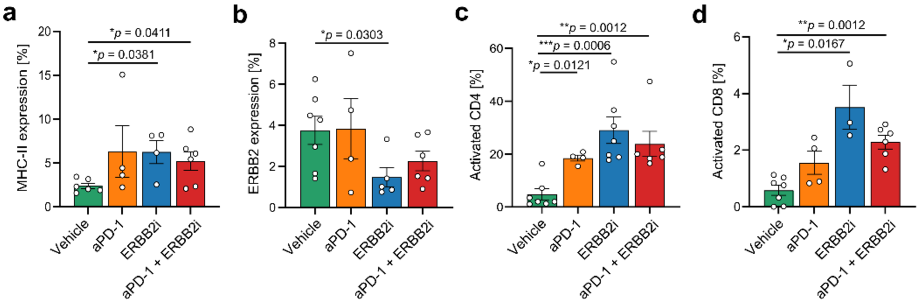

Supplementary Fig. 11: ERBB2 inhibition combined with anti-PD-1 blockade enhances MHC-II expression and reduces ERBB2 expression in mice suffering from SCLC. a-d, Marker expression on tumor cells from the lungs of autochthonous SCLC mice treated with vehicle, anti-PD-1 only, ERBB2i (mubritinib) only or anti-PD-1+ ERBB2i determined by flow cytometry. **a,** MHC-II (two-sided Mann–Whitney test; vehicle, n = 6; anti-PD-1, n = 4; ERBB2i, n = 4; anti-PD-1+ ERBB2i, n = 6 mice) determined by flow cytometry. **b,** ERBB2 expression (vehicle, n = 7; anti-PD-1, n = 4; ERBB2i, n = 5; anti-PD-1+ ERBB2i, n = 6 mice). **c,** Activated CD4 (CD4+ CD107a+) T cells (vehicle, n = 7; anti-PD-1, n = 4; ERBB2i, n = 7; anti-PD-1+ ERBB2i, n = 6 mice). **d,** Activated CD8 (CD8a+ CD107a+) T cells (vehicle group, n = 7; anti-PD-1, n = 4; ERBB2i, n = 3; anti-PD-1+ ERBB2i, n = 6 mice). Statistical analysis was performed using a two-sided Mann–Whitney test. Data are represented as mean ± SEM. **p* < 0.05, **p < 0.01, ***, p < 0.001. Source data are provided as a Source Data file.
