# Supplementary material for: ERBB2 signaling drives immune cell evasion and resistance against immunotherapy in small cell lung cancer
Source: Nat Commun. 2025 Dec 9;16:10983. doi: 10.1038/s41467-025-66800-x (PMC12689756; doi:10.1038/s41467-025-66800-x)
Supplement: Supplementary file 2 — Description of Additional Supplementary Files [file 41467_2025_66800_MOESM2_ESM.pdf]

Title: Supplementary Data 1

Description: [Potentially protein affecting mutations identified in SCLC metastatic cell line by whole-exome sequencing.]

Title: Supplementary Data 2

Description: [Potentially protein affecting mutations identified in SCLC primary cell line by whole-exome sequencing.]

Title: Supplementary Data 3

Description: [Modified phosphosites detected in phospho-protein profiling.]

Title: Supplementary Data 4

Description: [Potential relevance of detected phosphosites from Supplementary table 3.]

Title: Supplementary Data 5

Description: [Normalized gene expression in counts per million determined by RNA sequencing of WT and ERBB2 KO SCLC cells.]

Title: Supplementary Data 6

Description: [Clonotype distribution and T cell receptor sequencing of ERBB2i (mubritinib) + anti-PD-1 treated autochthonous SCLC mouse.]

Title: Supplementary Data 7

Description: [Differential gene expression analysis of clusters in scRNA/TCR sequencing in ERBB2i (mubritinib) + anti-PD-1 treated autochthonous SCLC mouse. Differential gene expression between T cell clusters was analyzed using the 10x Genomics Cell Ranger pipeline and Loupe Browser statistical framework, which applies a likelihood ratio test on the UMI counts. P-values were adjusted for multiple comparisons using the Benjamini-Hochberg method.]

Title: Supplementary Data 8

Description: [Clonotype distribution and T cell receptor sequencing of vehicle treated autochthonous SCLC mouse.]

Title: Supplementary Data 9

Description: [Differential gene expression analysis of clusters in scRNA /TCR sequencing in vehicle treated autochthonous SCLC mouse. Gene expression was processed using the 10x Genomics Cell Ranger pipeline and analyzed in Loupe Browser. Differential expression between clusters was calculated using the default Cell Ranger

statistical framework (likelihood ratio test) and p-values were adjusted for multiple comparisons using the Benjamini-Hochberg method.]

Title: Supplementary Data 10

Description: [Clinicopathological characteristics of SCLC samples.]
