# Supplementary material for: ERBB2 signaling drives immune cell evasion and resistance against immunotherapy in small cell lung cancer
Source: Nat Commun. 2025 Dec 9;16:10983. doi: 10.1038/s41467-025-66800-x (PMC12689756; doi:10.1038/s41467-025-66800-x)
Supplement: Supplementary file 13 — Reporting Summary [file 41467_2025_66800_MOESM13_ESM.pdf]

nature portfolio

Corresponding author(s): Roland T. Ullrich, Lydia Meder

Last updated by author(s): Oct 29, 2025

Reporting Summary

Nature Portfolio wishes to improve the reproducibility of the work that we publish. This form provides structure for consistency and transparency in reporting. For further information on Nature Portfolio policies, see our Editorial Policies and the Editorial Policy Checklist.

Statistics

For all statistical analyses, confirm that the following items are present in the figure legend, table legend, main text, or Methods section.

n/a	Confirmed	
☐	☒	The exact sample size (*n*) for each experimental group/condition, given as a discrete number and unit of measurement
☐	☒	A statement on whether measurements were taken from distinct samples or whether the same sample was measured repeatedly
☐	☒	The statistical test(s) used AND whether they are one- or two-sided<br>*Only common tests should be described solely by name; describe more complex techniques in the Methods section.*
☐	☒	A description of all covariates tested
☐	☒	A description of any assumptions or corrections, such as tests of normality and adjustment for multiple comparisons
☐	☒	A full description of the statistical parameters including central tendency (e.g. means) or other basic estimates (e.g. regression coefficient) AND variation (e.g. standard deviation) or associated estimates of uncertainty (e.g. confidence intervals)
☐	☒	For null hypothesis testing, the test statistic (e.g. *F*, *t*, *r*) with confidence intervals, effect sizes, degrees of freedom and *P* value noted<br>*Give P values as exact values whenever suitable.*
☒	☐	For Bayesian analysis, information on the choice of priors and Markov chain Monte Carlo settings
☒	☐	For hierarchical and complex designs, identification of the appropriate level for tests and full reporting of outcomes
☒	☐	Estimates of effect sizes (e.g. Cohen's *d*, Pearson's *r*), indicating how they were calculated

Our web collection on statistics for biologists contains articles on many of the points above.

Software and code

Policy information about availability of computer code

Data collection

Mass Spec: Samples were diluted with TEAB 50 mM to achieve a final concentration of Urea ≤ 2M. Samples were digested with trypsin (Serva) at an enzyme:substrate ratio of 1:75 and incubated at 25°C overnight. The enzymatic digestion was stopped by addition of a 1% solution of formic acid (Honeywell/FLUKA). 150 µL of suspended peptide samples were applied to the equilibrated TiO2 Spin Tip (Thermo Scientific). After centrifugation, the samples were re-applied to the Spin Tip in the microcentrifuge tube. The columns were washed by adding 20 µL of Binding/Equilibration Buffer and then washed by adding 20 µL of Wash Buffer. Finally, the columns were washed by adding 20 µL of LC-MS grade water (Merck). Excess liquid was removed by blotting the bottom of the spin tip onto a clean laboratory tissue and 50 µL of phosphopeptide elution buffer (Fischer Scientific) was added to the spin tip. The eluates were then immediately dried in a high-speed vacuum concentrator to remove the phosphopeptide elution buffer. The eluates were suspended in 50 µL of 0.1% formic acid for peptide concentration measurements using the Pierce™ Quantitative Colorimetric Peptide Assay Kit or direct MS analysis.

For proteome profiling performed with the DKTK Proteomics Core Facility (Goethe University Frankfurt, Germany), cell pellets were lysed in urea lysis buffer (8 M urea, 20 mM HEPES, pH 8.0, 1 mM sodium orthovanadate, 2.5 mM sodium pyrophosphate, 1 mM beta-glycerophosphate). Protein concentrations of the lysates were determined using the 660 nm assay kit (Thermo Fisher Scientific) according to the manufacturer's instructions. 800 µg protein per sample were reduced with DTT (10 mM for 1 h at 37 °C), alkylated with iodoacetamide (25 mM for 15 min at 37 °C in the dark) and digested using Lys-C (Wako/Fujifilm) for 2 h at 37 °C in an enzyme-to-substrate ratio of 1:50 (w/w). After dilution with 20 mM HEPES (pH 8.0) to a concentration of 2 M urea, digestion was continued overnight with trypsin (Promega) at 37 °C and 1:50 (w/w) enzyme-to-substrate ratio. The peptide mixtures were acidified and purified using C18 spin tips (Havard). For global proteome analysis, 10 µg peptide were dried by vacuum centrifugation and then dissolved in 0.1 % formic acid (FA). Peptide concentrations were determined using a fluorometric peptide assay (Thermo Fisher Scientific). To enrich phosphopeptides, 400 µg peptides were bound to TiO2 columns using the High-Select TiO2 Phosphopeptide Enrichment Kit (Thermo Fisher Scientific). Collected phosphopeptides from the eluate were also dried and resuspended in 0.1% FA. The peptide samples were analyzed by LC-MS/MS on a Vanquish Neo UHPLC system (Thermo Fisher Scientific) coupled online to an Orbitrap Astral mass spectrometer (Thermo Fisher Scientific) in a data-independent acquisition scheme

(DIA). 400 ng of peptides from each sample were concentrated and desalted on a PepMap Neo trap cartridge (Thermo Fisher Scientific, particle size 100 Å, inner diameter 300 µm, length 5 mm), followed by separation on a 15 cm PepMapNeo analytical column (Thermo Fisher Scientific) using a 30 min method (27 min linear gradient) of 1% to 28% acetonitrile in 0.1% formic acid at a flow rate of 800 nl/min. Precursor ion survey scans were acquired using the Orbitrap mass analyzer with the following parameters: resolution 240,000, scan range m/z 380-980, automatic gain control (AGC) target 5x10^6, maximum injection time 10 ms, RF lens setting 40%. For fragment ion scans using the Astral mass analyzer, precursor ions were isolated for collision-induced dissociation (HCD) through each survey scan with an isolation window of m/z 2, resulting in 299 scan events. The normalized HCD collision energy was set to 25% and for fragment ion analysis the AGC target was 5x10^4 at a maximum injection time of 3 ms.

Sequencing: Libraries of 3'mRNA were obtained from total RNA using the Lexogen QuantSeq kit according to standard protocol. After validation and quantification (2200 TapeStation, Agilent Technologies, Santa Clara, CA, USA and Qubit System, Invitrogen, Carlsbad, California, CA, USA respectively), pools of cDNA libraries were generated. Pools were quantified using the KAPA Library Quantification kit (Peqlab, Radnor, PA, USA) and the 7900HT Sequence Detection System (Applied Biosystems, Foster City, PA, USA) and lastly sequenced on an Illumina HiSeq4000 or NovaSeq6000 sequencer using a 2 × 100 base pair protocol.

scRNASeq: Single-cell RNA sequencing of tumor tissue was performed by Singleron Biotechnologies GmbH (Cologne, Germany). Murine SCLC tumor samples were harvested from lungs of treated mice, washed in PBS, covered by Sample Preparation Buffer (Singleron Biotechnologies) and shipped on ice. Samples were processed within 72 hours after tumor isolation using sCelLiVETM Tissue Dissociation Buffer (Singleron Biotechnologies) and microfluidic SCOPE-chipTM. Barcode hybridization was followed by reverse transcription and cDNA amplification. Amplified cDNA was fragmented, ligated to adapters and PCR amplified to construct a sequencing library suitable for Illumina based sequencing.

Data analysis

Mass Spectometry: Samples were analyzed by the Cellular Stress Responses in Aging-Associated Diseases (CECAD) Proteomics Core Facility (University of Cologne, Germany) on an Orbitrap Exploris 480 mass spectrometer (Thermo Scientific) coupled to an Evosep ONE (Evosep). The Evosep was run with its Whisper Zoom 20 SPD gradient using an Aurora Elite pulled-tip column (Ionopticks). The mass spectrometer was operated using a WHISH-DIA approach39. MS2 spectra were acquired in the range of 400 to 1000 m/z at 60k resolution in 25 m/z windows, resulting in 24 scans total. Fragments were acquired in a range of 250 to 1500 m/z with a normalized AGC target of 1000% and 30% normalized HCD collision energy. Every 6 scans, an MS1 scan was inserted, which was acquired at a resolution of 120k in the range of 390 – 1010 m/z. Samples were analyzed in Spectronaut 19 (Biognosys) using standard setting for directDIA analysis, but quantification performed on MS1 level and requiring at least 6 fragment ions. Results were searched against the canonical murine Uniprot reference proteome (UP589, downloaded 15/01/2025) with follow-up analysis performed in Perseus 1.6.1540.

Raw DIA data were analyzed using Proteome Discoverer (v.3.1.1.93, Thermo Fisher Scientific). Spectra were searched against the Uniprot mouse reference proteome and 245 frequently observed contaminants using the CHIMERYS search algorithm. The mass tolerance for fragment ions was set to 10 ppm. Oxidation of methionine was considered as dynamic modification while carbamidomethylation of cysteine was defined as a fixed modification. The peptide length was defined as between seven to 30 amino acids with one allowed missed cleavage site. One to four charges per peptide were allowed. At both peptide and protein level, the false discovery rate (FDR) was set at 1%. For phosphoproteome analysis, Phospho Modifications (S, T, Y) were set as dynamic modification. Further data processing was done using R studio (v.2024.09.1). First, contaminants were removed. To control for equal sample loading, intensities from each LC-MSMS run were normalized on the median of the summed-up intensities from each sample41. Phosphoproteome analysis was performed at the site-specific level. Peptide abundances were merged by sequence with the modification sites. If more than one peptide sequence group matched a site (e.g. due to miscleavages), the abundance of all peptide sequence groups for the site was summed.

Sequencing: In brief, the FASTQ files were aligned to the ensembl GRCm39 reference using Bwa v0.7.17 and Samtools v1.13. Duplicate reads were marked with Picard v2.26.0. Mutations were called using GATK Mutect2 v4.2.1.0 in tumor only mode. The panel of normal for this analysis included 14 healthy murine samples from our previous study37. The mutations were filtered using GATK FilterMutectCalls v4.2.1.0 and annotated using ensemble-vep v113.4. Variants were excluded if present in the strain reference files from Wellcome Sanger Mouse Genome Project (mgp_REL2021_indels and mgp_REL2021_snps) or in the ensembl variation database v113.4 or if they were predicted not to affect protein sequence. Exon coverage was collected using GATK CollectReadCounts v4.2.1.0 and normalized to reads / million.

scRNASeq: For single-cell transcriptome analysis, quality control was performed prior to downstream analysis. CeleScope was used to generate a single-cell gene expression matrix file based on the raw sequencing data. Standard single-cell gene expression QC metrics were used to identify high quality single cells removing doublets (large gene or Unique Molecular Identifier - UMI counts), dying cells (measuring mitochondrial RNA reads) and debris cells (with small UMI counts). For cell clustering and visualization in 2 dimensions (UMAP), annotation of cell types, cell type differential gene expression analysis and visualization, we utilized the Scanpy Python package41, a robust toolkit designed for single-cell data analysis, to perform quality control, ensuring inclusion of cells with over 150 genes. Additionally, cells exhibiting high mitochondrial and ribosomal gene content were excluded. We detected and removed potential doublets using a semi-supervised deep learning method42. The expression matrix was globally scaled by normalizing gene expression measurements relative to the total expression per cell. Thereafter, the resulting values were scaled by a factor of 10,000 followed by a natural logarithm transformation with a pseudocount value of 1. Principal component analysis (PCA) was used to find a lower-dimensional representation of the data. The top 50 principal components (PCs) were used for global clustering and for visualization using a UMAP43. Clusters were identified using the Phenograph-louvain44 clustering algorithm integrated in Scanpy. This step resulted in a total of 30 global clusters. The analyses were performed in Python using Scanpy and anndata. UMAP plots were created and visualized using Scanpy.

For manuscripts utilizing custom algorithms or software that are central to the research but not yet described in published literature, software must be made available to editors and reviewers. We strongly encourage code deposition in a community repository (e.g. GitHub). See the Nature Portfolio guidelines for submitting code & software for further information.

Data

Policy information about availability of data

All manuscripts must include a data availability statement. This statement should provide the following information, where applicable:

- Accession codes, unique identifiers, or web links for publicly available datasets
- A description of any restrictions on data availability
- For clinical datasets or third party data, please ensure that the statement adheres to our policy

Sequencing and proteomic data generated in this study are publicly available in the following MINSEQE-compliant repositories: Proteomic data are available in the PRIDE database (ID PXD065735) [https://www.ebi.ac.uk/pride/archive/projects/PXD065735] and in the MassIVE repository (ID PXD066359) [https://proteomecentral.proteomexchange.org/cgi/GetDataset?ID=PXD066359]. Whole-exome sequencing data have been deposited in the Sequence Read Archive (SRA) (BioProject ID PRJNA1293554) [https://www.ncbi.nlm.nih.gov/bioproject/PRJNA1293554]. TCR sequencing data are available in the Gene Expression Omnibus (GEO)

under accession number GSE282715 [https://www.ncbi.nlm.nih.gov/geo/query/acc.cgi?acc=GSE282715]. Bulk RNA sequencing data are available in GEO under GSE283573 [https://www.ncbi.nlm.nih.gov/geo/query/acc.cgi?acc=GSE283573] and GSE303491 [https://www.ncbi.nlm.nih.gov/geo/query/acc.cgi?acc=GSE303491], and single-cell RNA sequencing data under GSE283827 [https://www.ncbi.nlm.nih.gov/geo/query/acc.cgi?acc=GSE283827]. Additional materials are available upon request from the corresponding authors. The single-cell RNA-seq data from Chan et al.10 used for comparative analysis were accessed via Cell x Gene [https://cellxgene.cziscience.com/e/34deb33b-a50e-4993-a38b-1c0e5079c1c2.cxg]. RNA expression data from human SCLC cell lines were obtained from the GDSC-MGH-Sanger dataset accessed via CellMinerCDB [https://discover.nci.nih.gov/cellminercdb/]. The IMpower133 dataset was accessed from the European Genome-phenome Archive under the identifier EGAS50000000138 via https://ega-archive.org/ with the approval DA01145. These data were not generated in this study. Source data are provided with this paper. The remaining data are available within the Article, Supplementary Information or Source Data file.

Research involving human participants, their data, or biological material

Policy information about studies with human participants or human data. See also policy information about sex, gender (identity/presentation), and sexual orientation and race, ethnicity and racism.

Reporting on sex and gender	Reporting of sexes of SCLC patients is done in the Supplementary Table 10.
Reporting on race, ethnicity, or other socially relevant groupings	We do not report race, ethnicity or social relevant groupings.
Population characteristics	n/a
Recruitment	n/a
Ethics oversight	All human subject research was performed in strict accordance with approved protocols by the local ethics committee of the University of Cologne and with the recognized ethical guidelines of the Declaration of Helsinki. Tumor tissue (reference no. 13-091) was obtained during routine clinical procedures from lung cancer patients providing written informed consent.

Note that full information on the approval of the study protocol must also be provided in the manuscript.

Field-specific reporting

Please select the one below that is the best fit for your research. If you are not sure, read the appropriate sections before making your selection.

☒ Life sciences ☐ Behavioural & social sciences ☐ Ecological, evolutionary & environmental sciences

For a reference copy of the document with all sections, see nature.com/documents/nr-reporting-summary-flat.pdf

Life sciences study design

All studies must disclose on these points even when the disclosure is negative.

Sample size	Sample sizes are chosen based on included and approved animal numbers.
Data exclusions	Cells in scRNA Seq exhibiting high mitochondrial and ribosomal gene content were excluded.
Replication	In vitro and in vivo replicates have been performed as indicated. In vivo replicates have been limited by approved animal numbers.
Randomization	Male and female mice have been randomized into the groups before tumor induction.
Blinding	Blinding has not been performed.

Reporting for specific materials, systems and methods

We require information from authors about some types of materials, experimental systems and methods used in many studies. Here, indicate whether each material, system or method listed is relevant to your study. If you are not sure if a list item applies to your research, read the appropriate section before selecting a response.

Materials & experimental systems

n/a	Involved in the study	
☐	☒	Antibodies
☐	☒	Eukaryotic cell lines
☒	☐	Palaeontology and archaeology
☐	☒	Animals and other organisms
☐	☒	Clinical data
☒	☐	Dual use research of concern
☒	☐	Plants

Methods

n/a	Involved in the study	
☒	☐	ChIP-seq
☐	☒	Flow cytometry
☒	☐	MRI-based neuroimaging

Antibodies

Antibodies used | Western Blot: (pERBB2, Cell signaling, Cat. #2243; pTBK1, Cell signaling, Cat. #5483; pERK1/2, Cell signalling Cat. #9106; pAKT, Cell signalling, Cat. #9271; ERBB2, Cell signalling, Cat. #4290; TBK1, Cell signalling, Cat. #3504; ERK1/2, Cell signalling; Cat. #9102; AKT, Cell signalling, Cat. #9272; Rig-I (D14G6), Cell signalling, Cat. #3743; cGas (D3080), Cell signalling, Cat. #31659; pSting, Invitrogen, Cat. #PA5-105674; Actin (Clone C4), MP, Cat. #691001); IHC: against MHC-I (abcam, clone EPR1394Y, Cat. # ab134189), ERBB2 (Roche, clone 4B5, Cat. # 790-2991) and NCAM-1 (Zytomed, Cat. #RBK050); FACS: CD3 (Alexa-Fluor-700, clone 17A2, Biolegend, Cat. #100216), CD4 (PE-Dazzle 594, clone GK1.5, Biolegend, Cat. # 100456), CD45 (APC-Cy7, clone 30-F11, Biolegend, Cat. # 103116), CTLA-4 (PE, UC10-4B9 Thermo Fisher, Cat. #14-1522-82), CD56 (APC, R&D Systems, clone 809220, Cat. # FAB7820A), CD8a (FITC, clone 53-6.7, Biolegend, Cat. # 100705; Pacific blue, clone 53-6.7, Biolegend, Cat. # 100728), H2Kb (Pacific Blue, clone AF6-88.5, Biolegend, Cat. # 116517), PD-1 (APC, clone 29F.1A12, Biolegend, Cat. # 135210), PD-L1 (PE-Cy7, clone 10F.9G2, Biolegend, Cat. # 124313), TIM-3 (PerCP-Cy5.5, clone B8.2C12, Biolegend, Cat. # 134011)

Validation | Primary antibodies are validated by the manufacturer.

Eukaryotic cell lines

Policy information about cell lines and Sex and Gender in Research

Cell line source(s) | Human lung cancer cell lines were kindly provided by Roman K. Thomas (Department of Translational Genomics, University of Cologne, Germany) and Reinhard Büttner (Institute for Pathology, University Hospital Cologne, Germany). Murine SCLC cell lines were established after harvesting primary SCLC lung tumors or liver metastasis of the autochthonous SCLC mouse model.

Authentication | Microsatellites are used for authentication of cell lines.

Mycoplasma contamination | Cell lines were tested for mycoplasma contamination every three months.

Commonly misidentified lines (See ICLAC register) | n/a

Animals and other research organisms

Policy information about studies involving animals; ARRIVE guidelines recommended for reporting animal research, and Sex and Gender in Research

Laboratory animals | The genetically engineered SCLC mouse model is driven by conditional deletion of the tumor suppressor genes Rb1 and Trp53, previously published PMID: 14522252 DOI: 10.1016/s1535-6108(03)00220-4. Other mouse strains include C57BL/6J and NOD.Cg-Prkdcscid Il2rgtm1Wjl/SzJ (NSG).

Wild animals | n/a

Reporting on sex | Male and female mice with a C57BL/6 background, a minimal age of 6 weeks and a minimal weight of 20 g are included in the study. Animals of both sexes were randomly assigned to experimental groups and sex was not considered in the study design or analysis.

Field-collected samples | n/a

Ethics oversight | Animal Experiments were performed in accordance with FELASA recommendations. The protocol was approved by the local animal welfare committee of the University of Cologne and authorized by the Landesamt für Natur, Umwelt und Verbraucherschutz (LANUV NRW, Düsseldorf) (TVA 2025-219; 84-02.04.2015.A199; 81-02.04.2020.A026; 81-02.04.2020.A219; 81-02.04.2020.A328). All procedures were conducted in accordance with institutional, national, and international guidelines.

Note that full information on the approval of the study protocol must also be provided in the manuscript.

Clinical data

Policy information about clinical studies

All manuscripts should comply with the ICMJE guidelines for publication of clinical research and a completed CONSORT checklist must be included with all submissions.

Clinical trial registration | n/a

Study protocol | 13-091

Data collection | FFPE materal of SCLC patients was collected in the Institute for Pathology, University Hospital Cologne, Germany.

Outcomes | Evaluation of IHC stain intensity for MHC-I and ERBB2.

Plants

Seed stocks	n/a
Novel plant genotypes	n/a
Authentication	n/a

Flow Cytometry

Plots

Confirm that:

- [x] The axis labels state the marker and fluorochrome used (e.g. CD4-FITC).
- [x] The axis scales are clearly visible. Include numbers along axes only for bottom left plot of group (a 'group' is an analysis of identical markers).
- [x] All plots are contour plots with outliers or pseudocolor plots.
- [x] A numerical value for number of cells or percentage (with statistics) is provided.

Methodology

Sample preparation	Tumour tissue was isolated by 40 µm cell strainer and red blood cells were lysed by ACK lysing Buffer. After washing with PBS, the cell suspension was incubated with the staining mix for 30min at 4°C.
Instrument	Flow cytometry measurements were performed on a Gallios 10/3 (Beckman Coulter).
Software	Data was analyzed using Kaluza (Beckman Coulter).
Cell population abundance	Cell population abundance is indicated as % of parental population.
Gating strategy	Gating strategies are provided.

- [x] Tick this box to confirm that a figure exemplifying the gating strategy is provided in the Supplementary Information.
