# Supplementary material for: ERBB2 signaling drives immune cell evasion and resistance against immunotherapy in small cell lung cancer
Source: Nat Commun. 2025 Dec 9;16:10983. doi: 10.1038/s41467-025-66800-x (PMC12689756; doi:10.1038/s41467-025-66800-x)

Fig 4a.

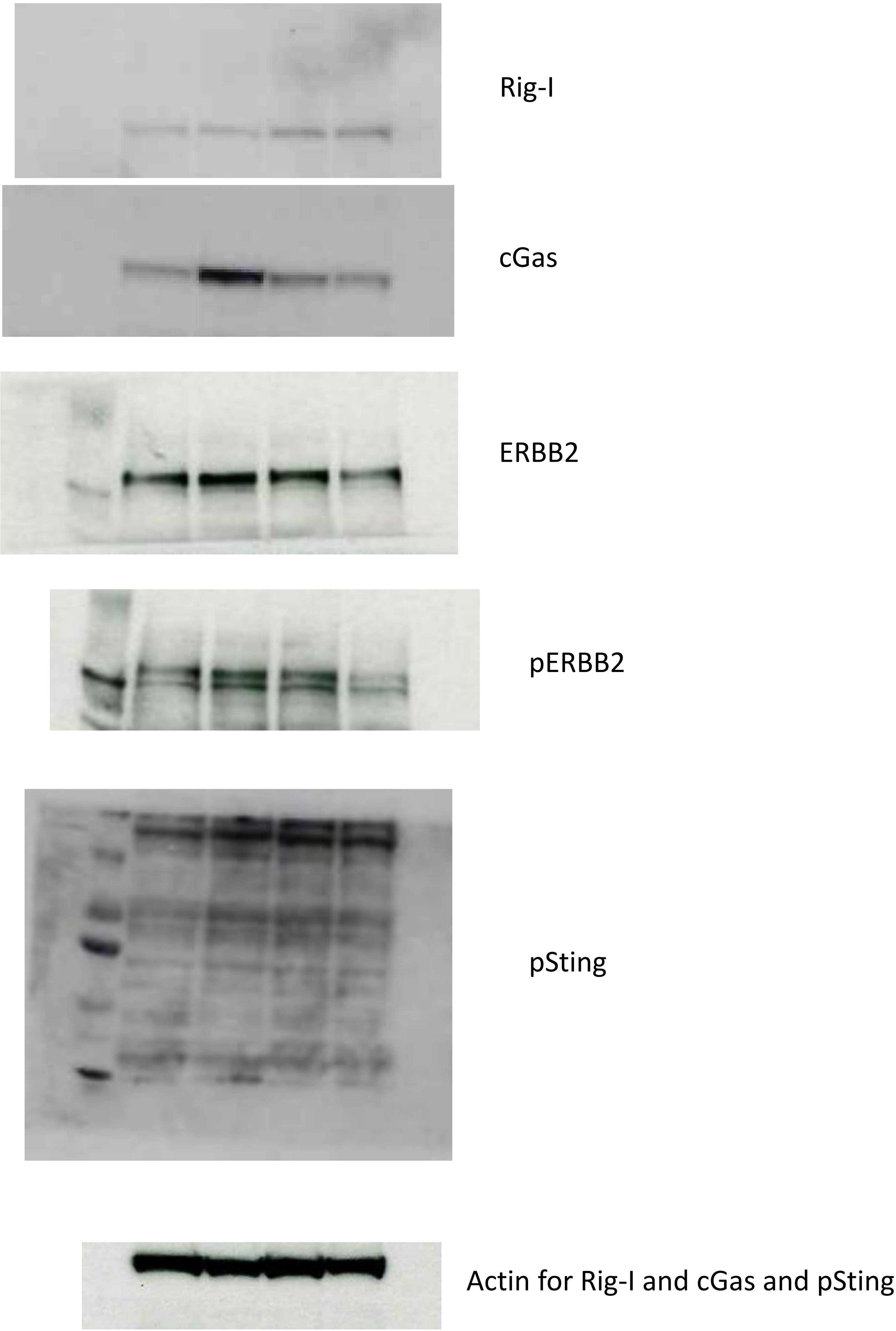
Rig-I
cGas
ERBB2
pERBB2
pSting
Actin for Rig-I and cGas and pSting

Fig 4a.

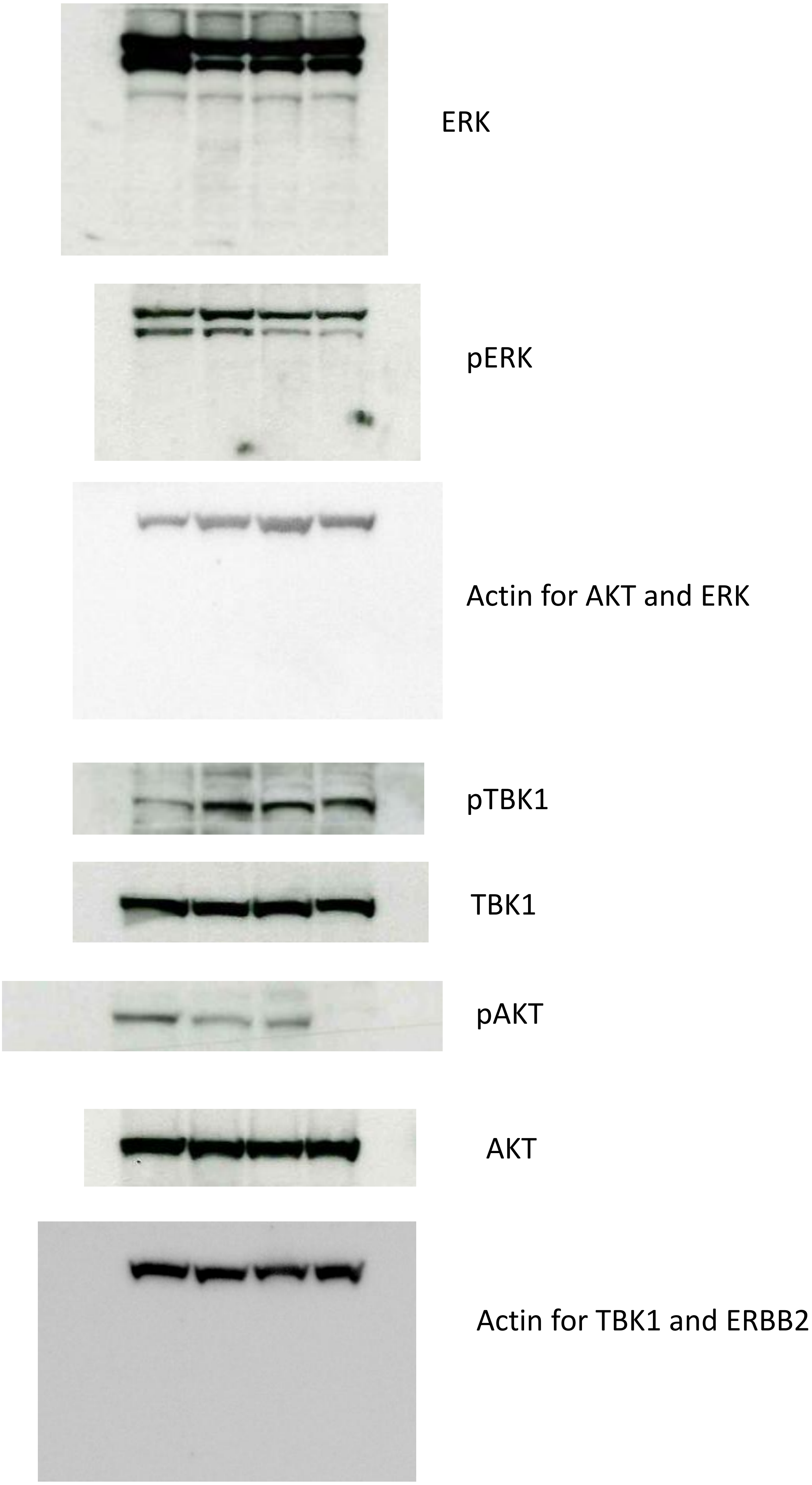
ERK
pERK
Actin for AKT and ERK
pTBK1
TBK1
pAKT
AKT
Actin for TBK1 and ERBB2

Fig 4b.

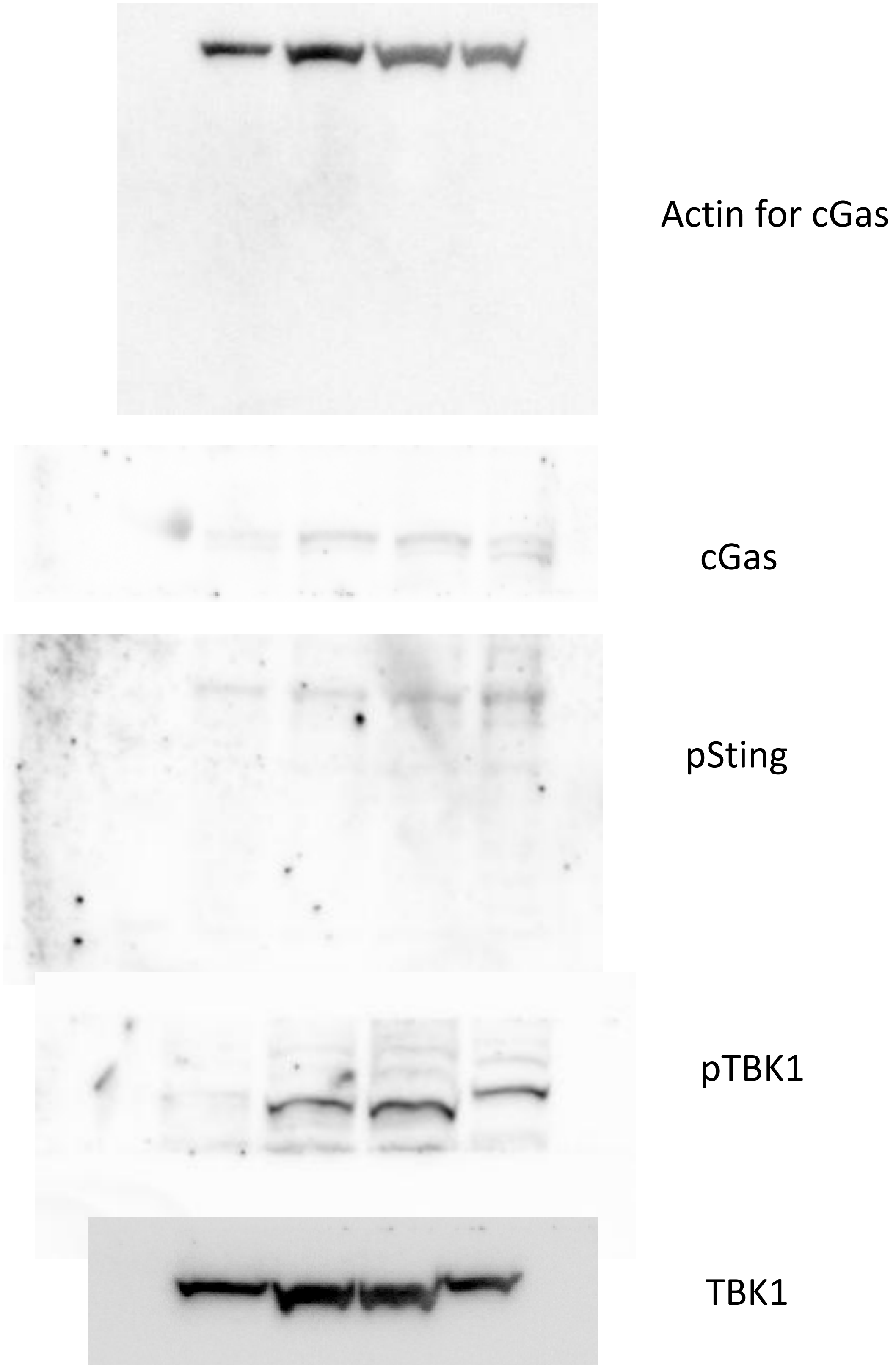

Fig 4c.

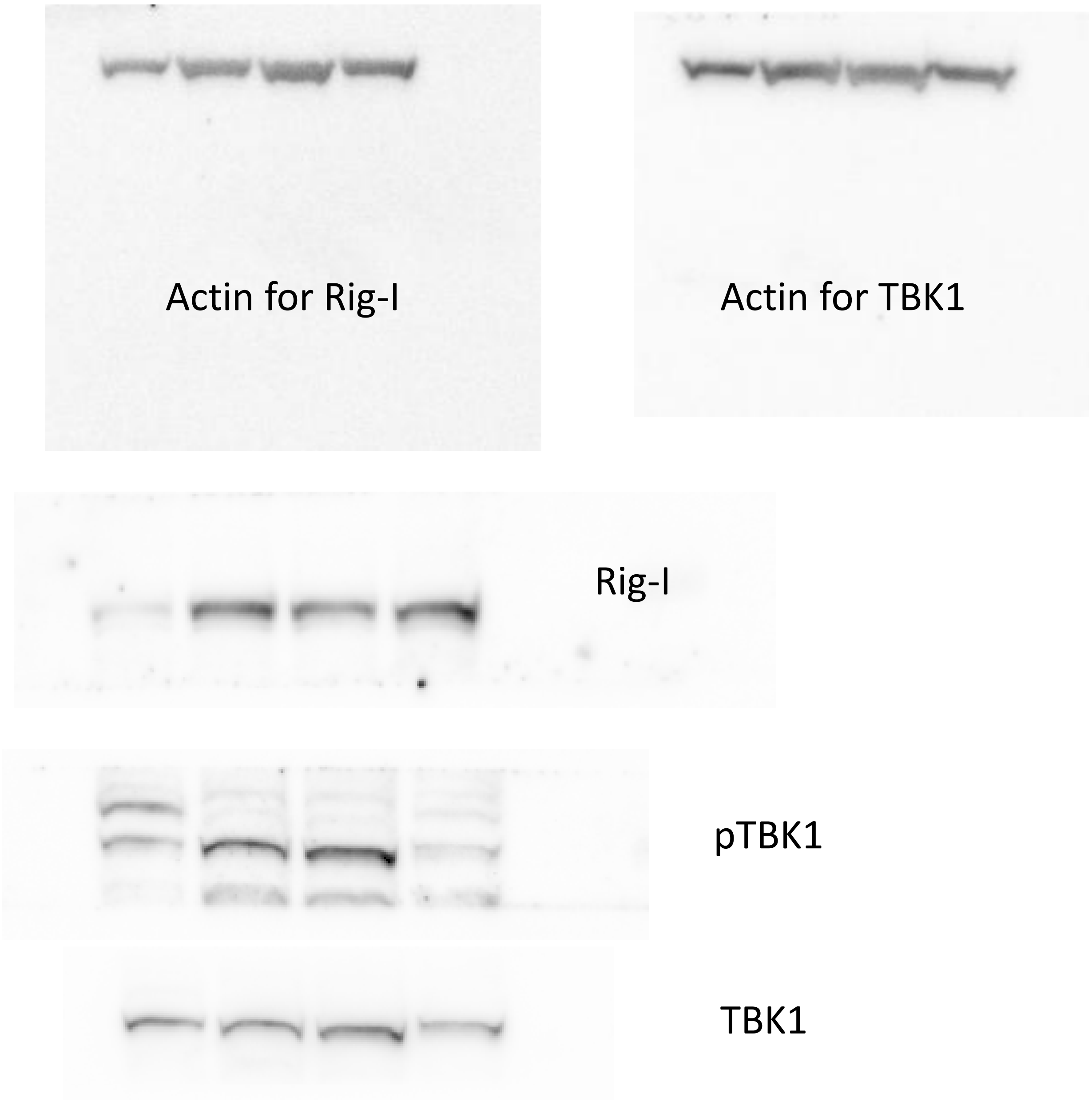

Supplementary Fig 3a.

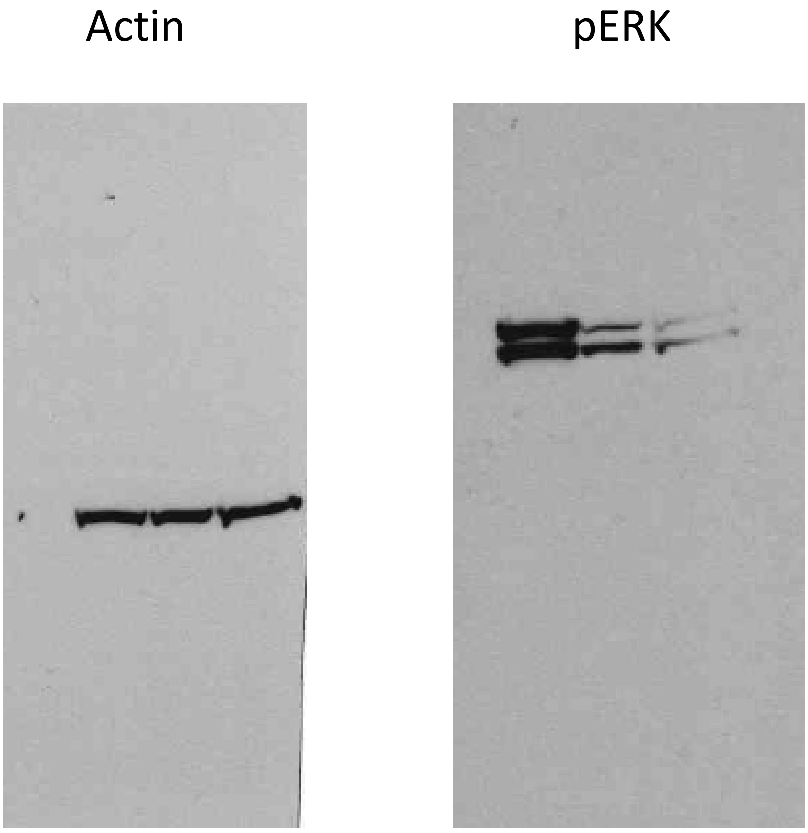

Supplementary Fig. 9a

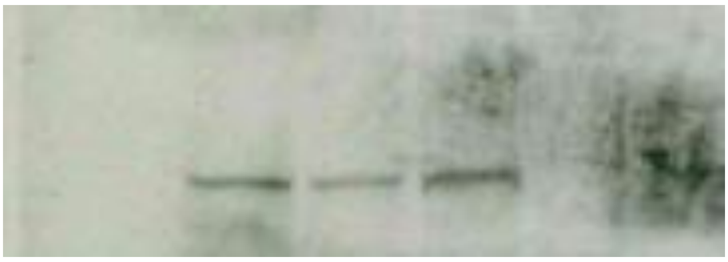

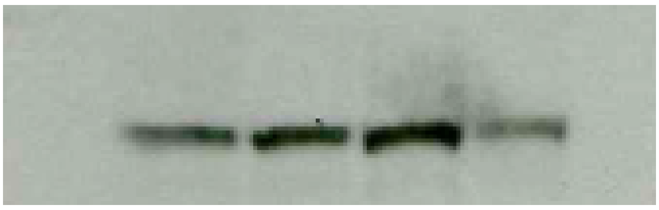

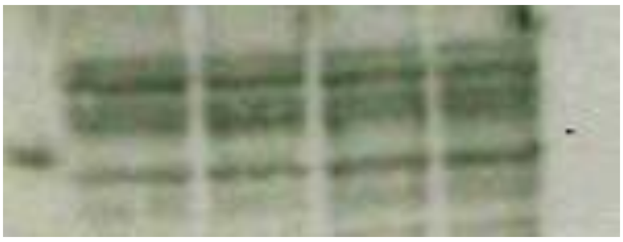

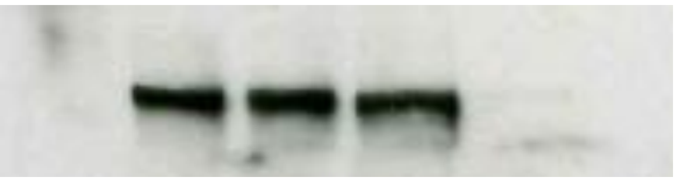

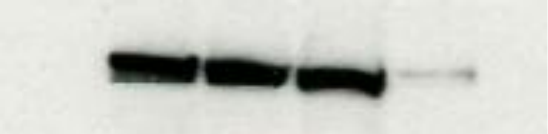

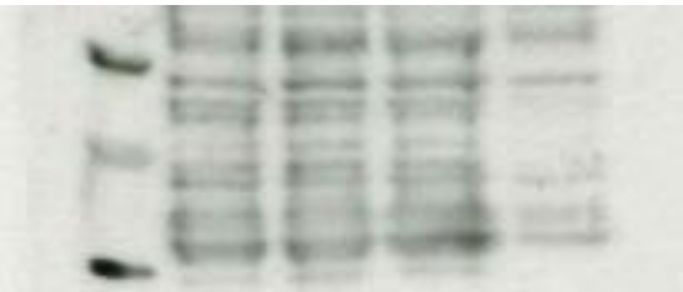

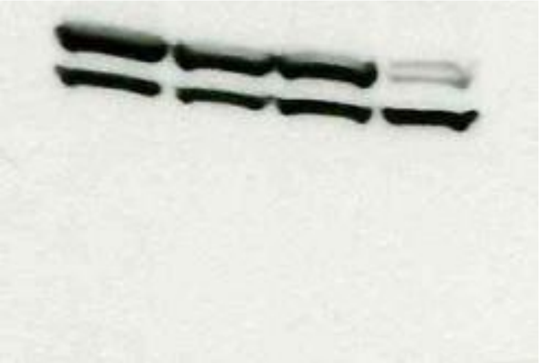

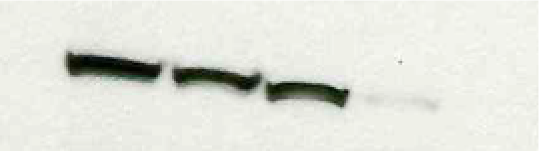

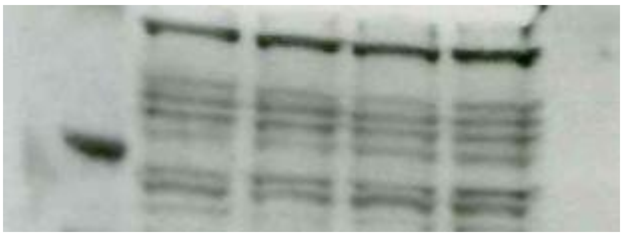

Supplementary Fig. 9a

phospho blots

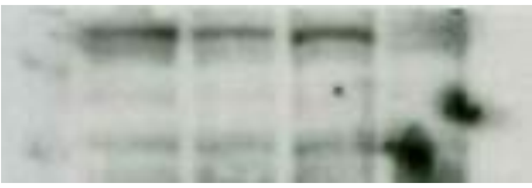

pSting

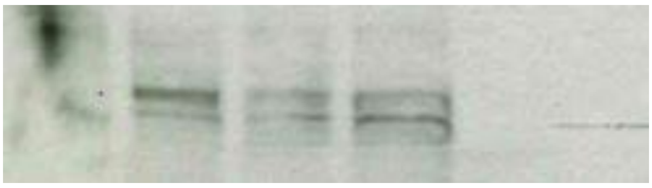

pERBB2

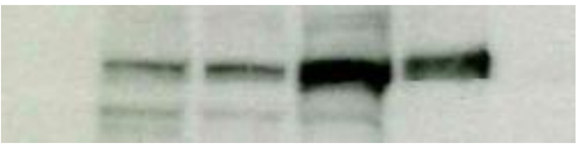

pTBK1

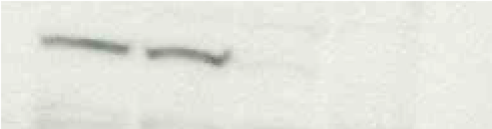

pAKT

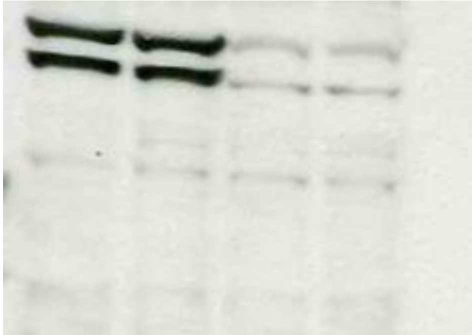

pERK

Supplementary Fig. 9b

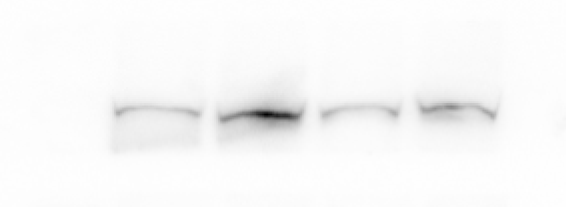

TBK1 with MK-2206 treatment

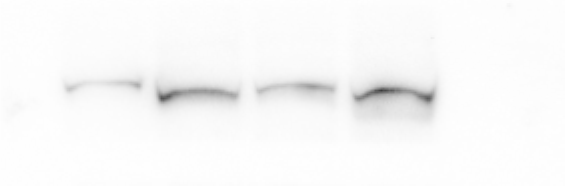

TBK1 with PD-0325901 treatment

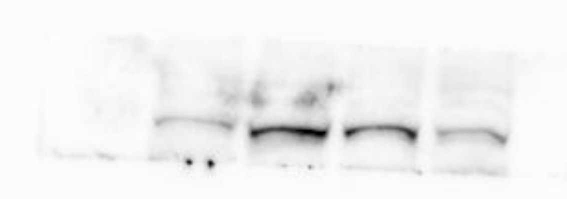

pTBK1 with MK-2206 treatment

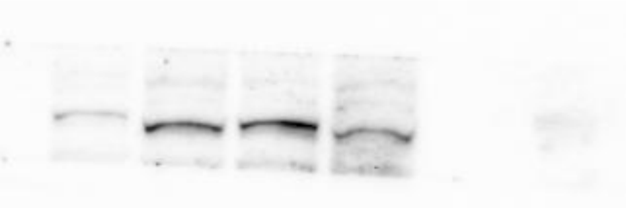

pTBK1 with PD-0325901 treatment

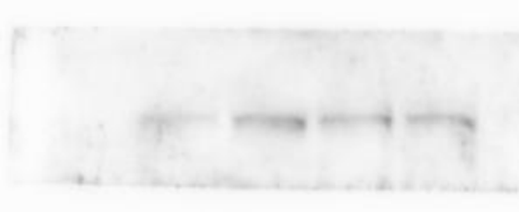

cGas

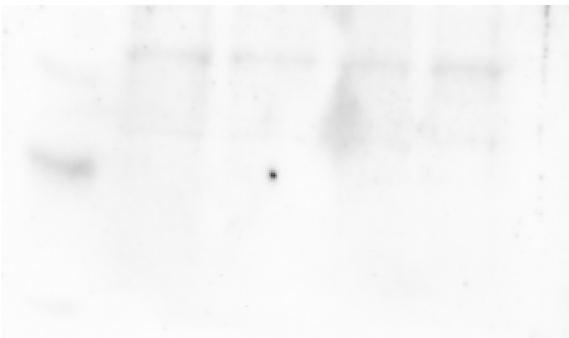

pSting

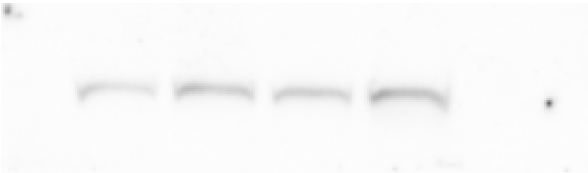

Rig-I

Supplementary Fig. 9b

Actin blots

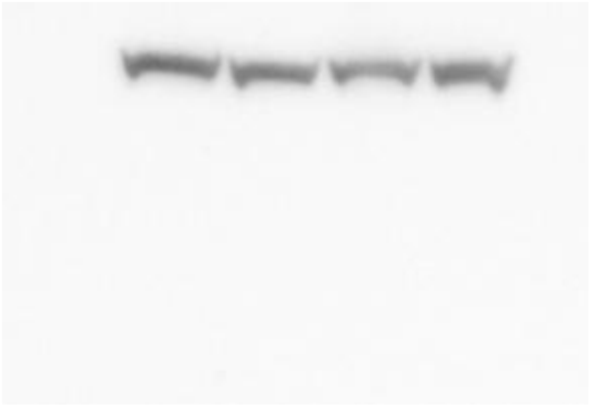

Actin for cGas and pSting

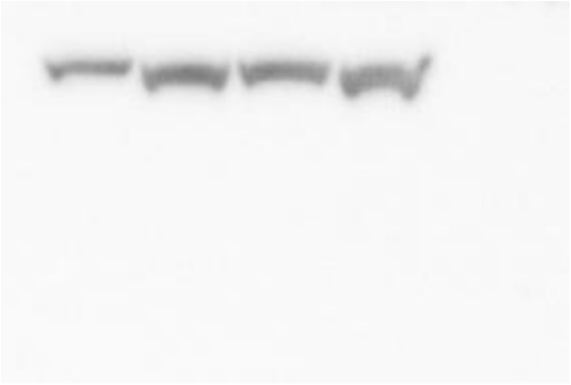

Actin for Rig-I

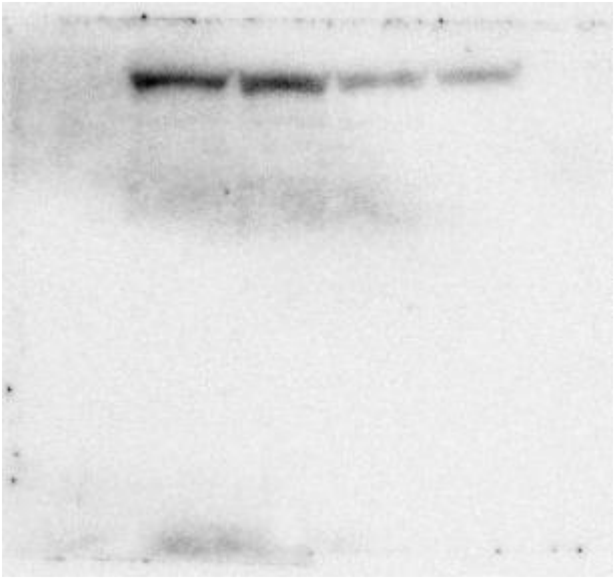

Actin for TBK1 with MK-2206 treatment

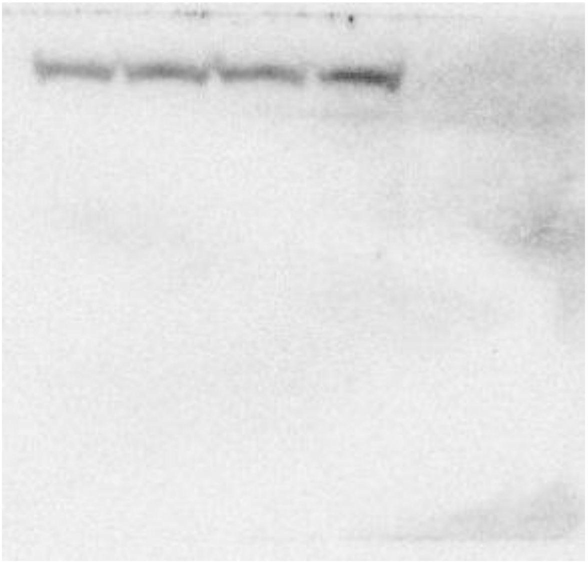

Actin for TBK1 with PD-0325901 treatment

Supplementary Fig. 9c

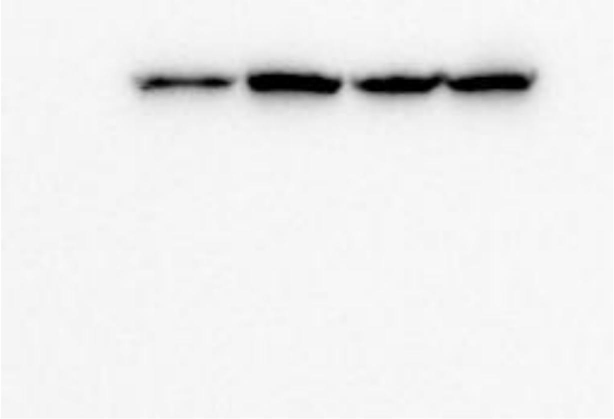

Actin for mSCLC M1

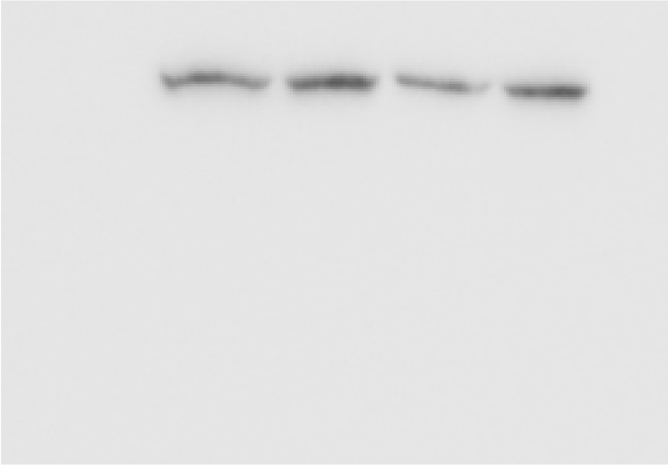

Actin for mSCLC P1

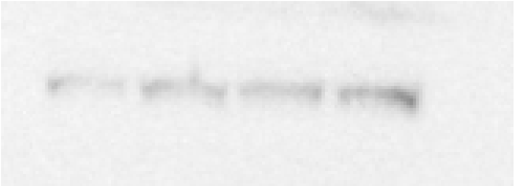

AKT for mSCLC M1

AKT for mSCLC P1

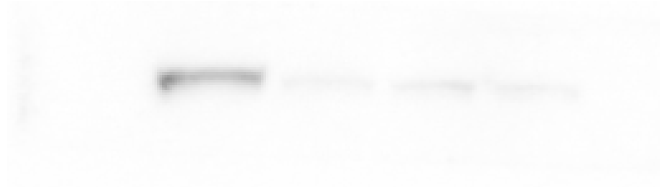

pAKT for mSCLC M1

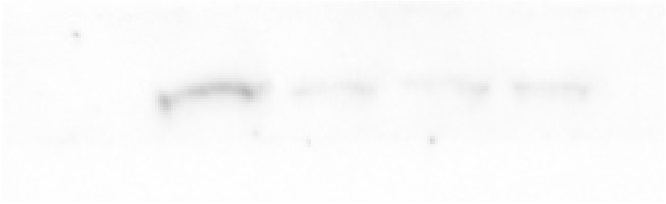

pAKT for mSCLC P1

Supplementary Fig. 9d

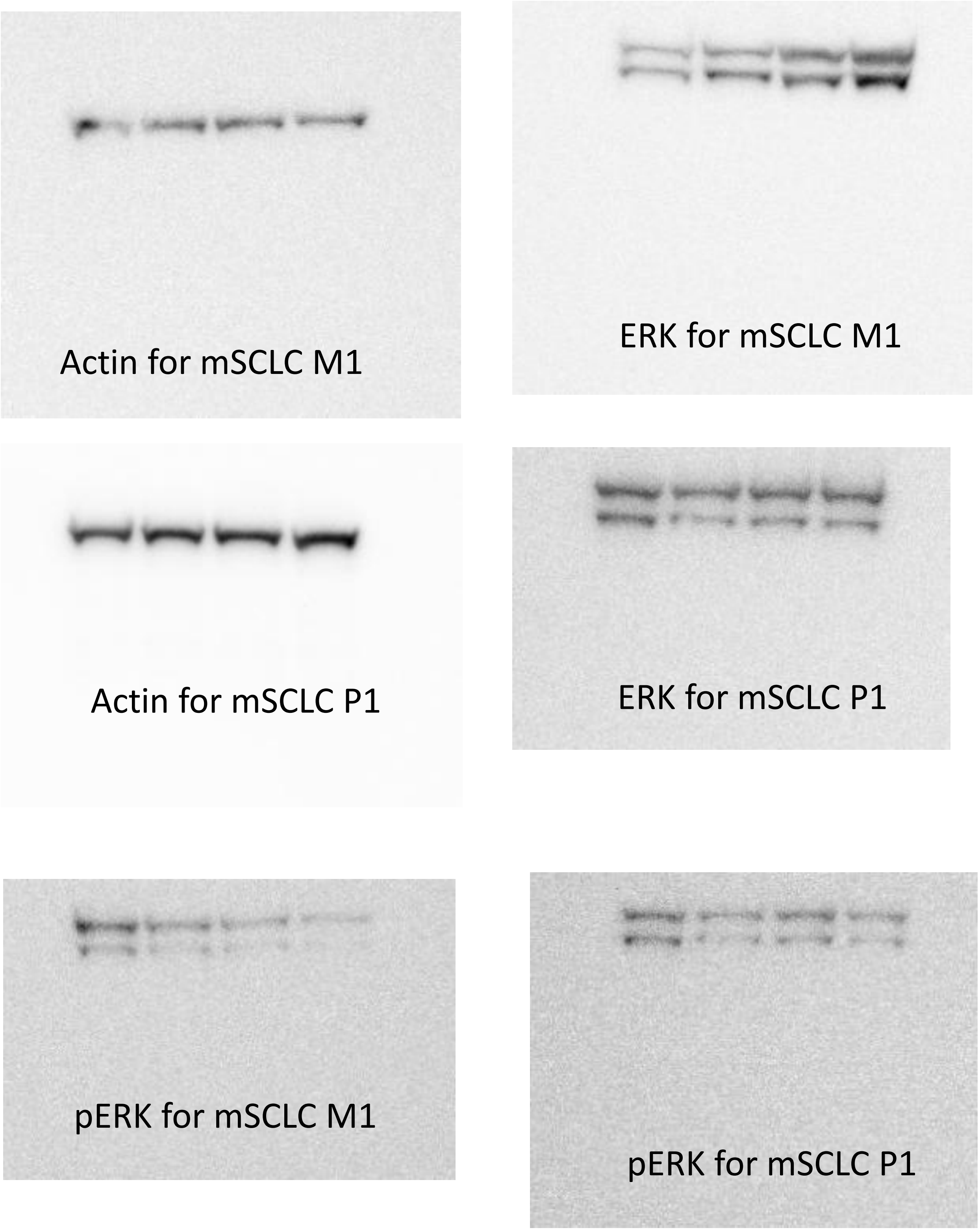

Supplementary Fig. 9e

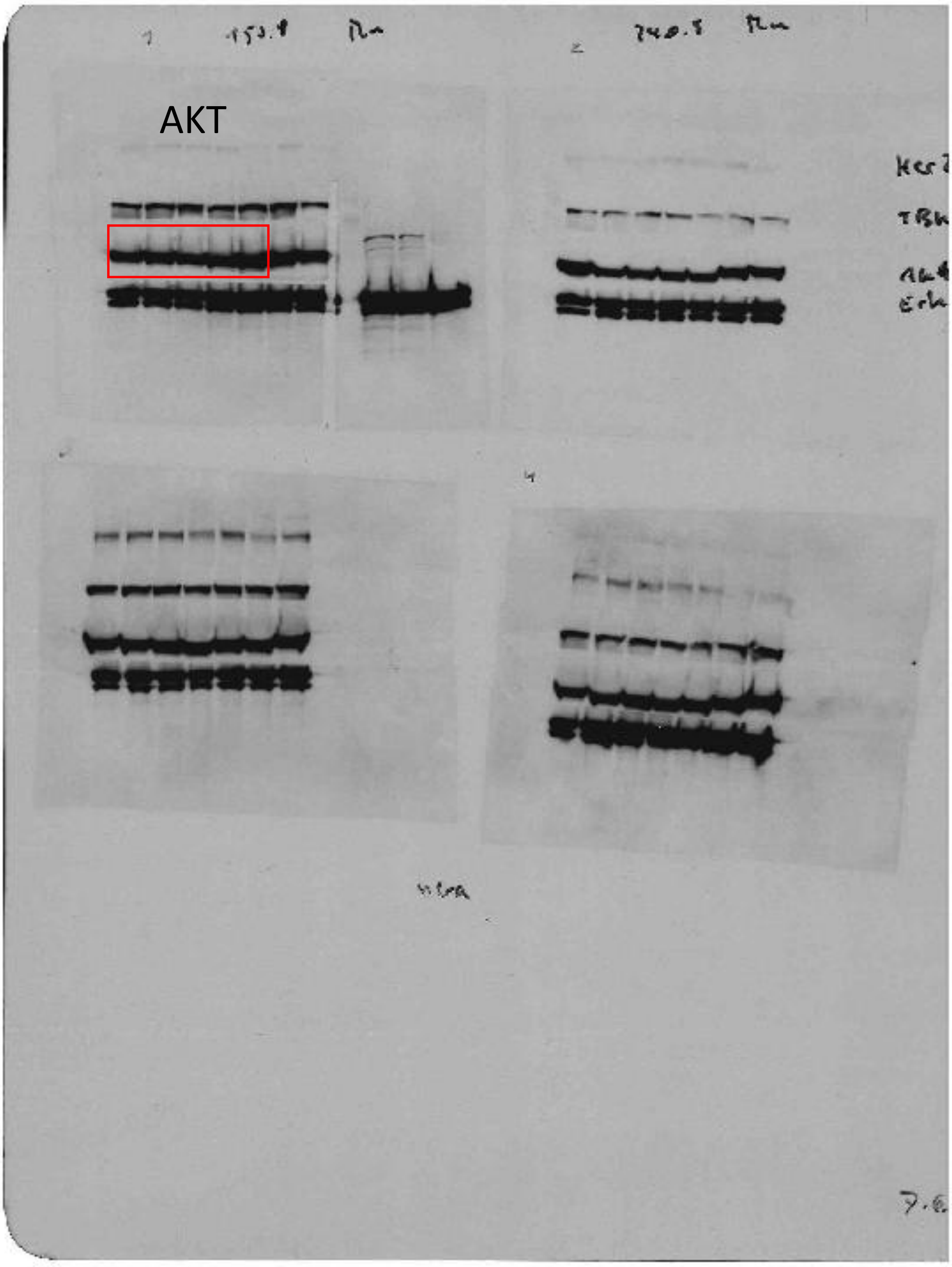

Supplementary Fig. 9e

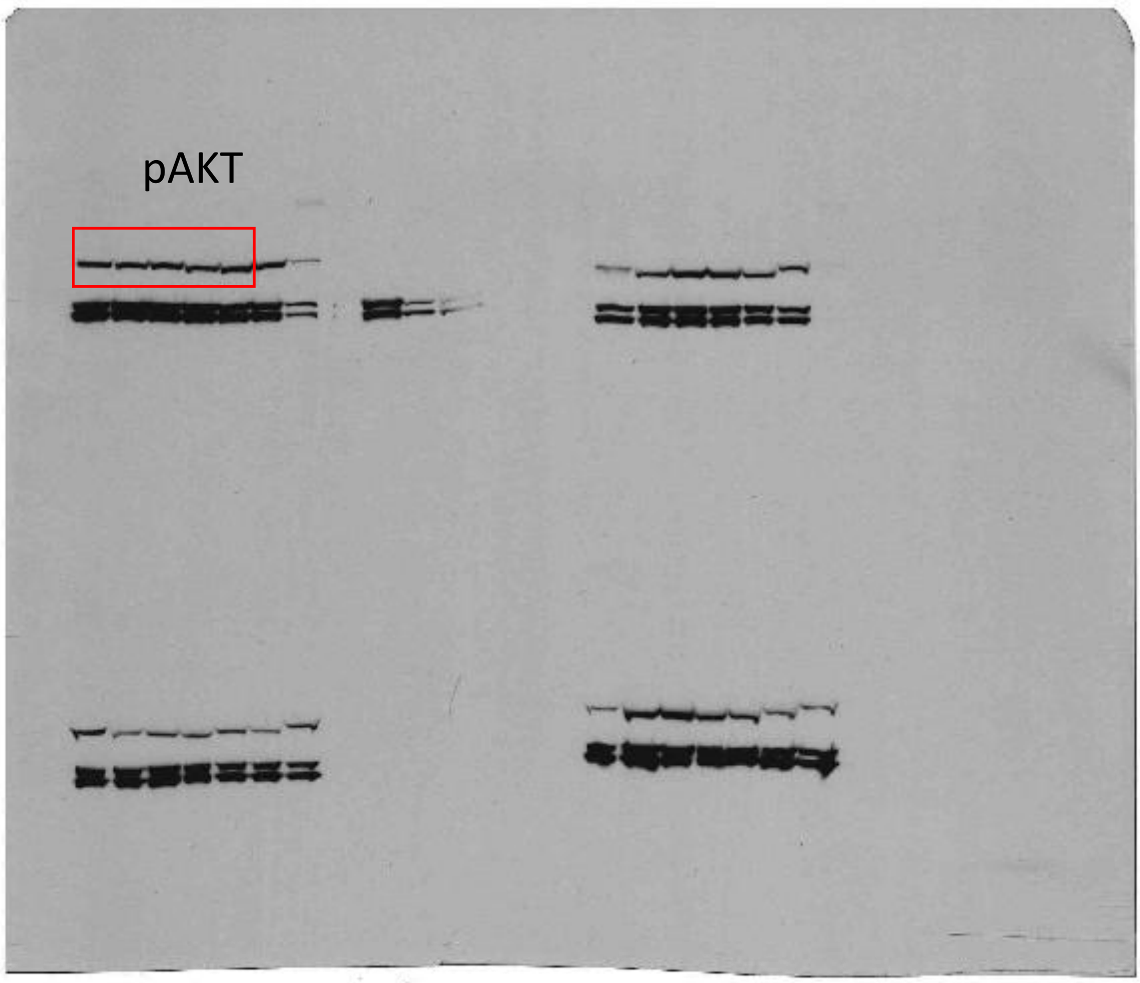

Supplementary Fig. 9e

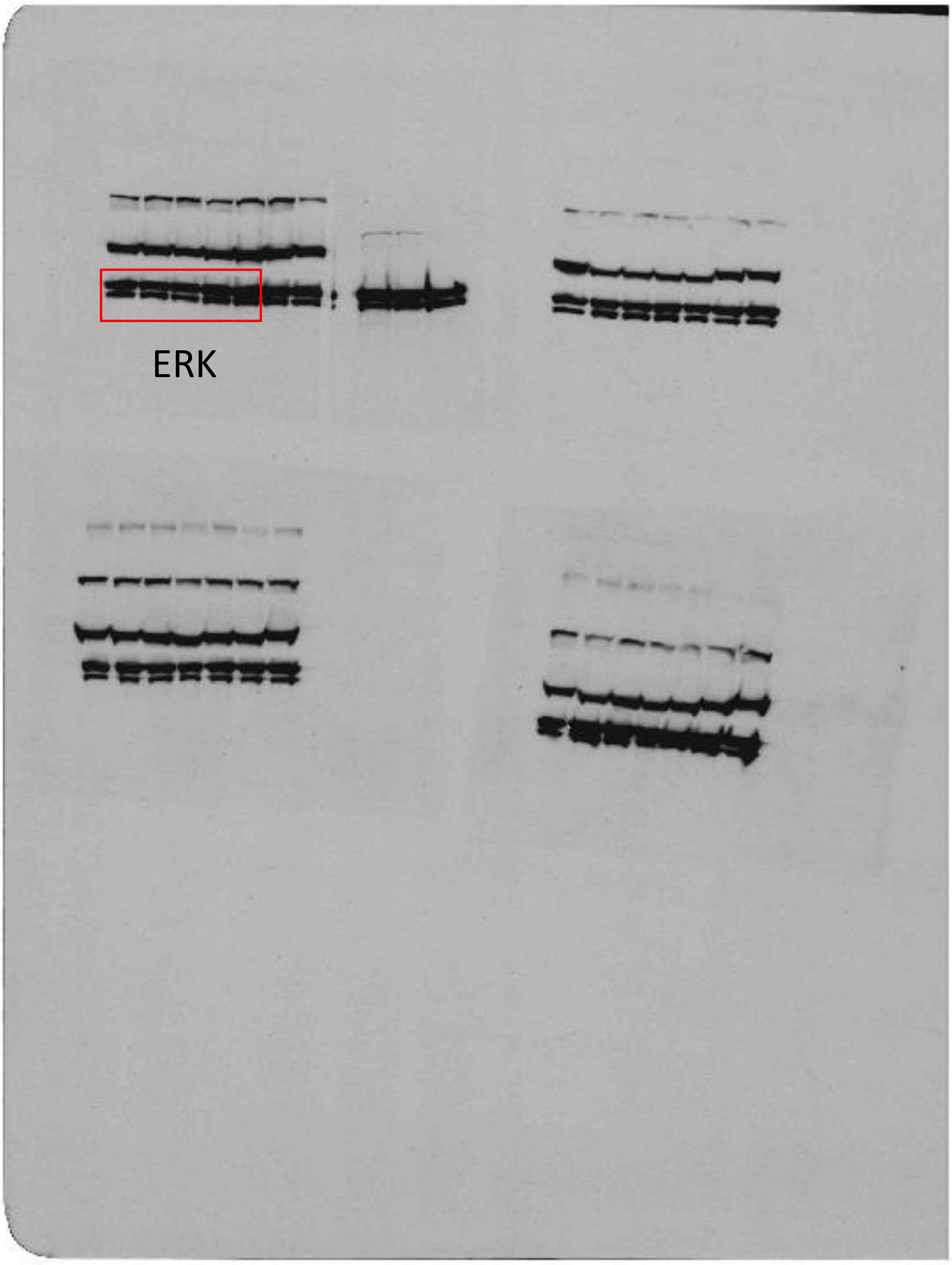

Supplementary Fig. 9e

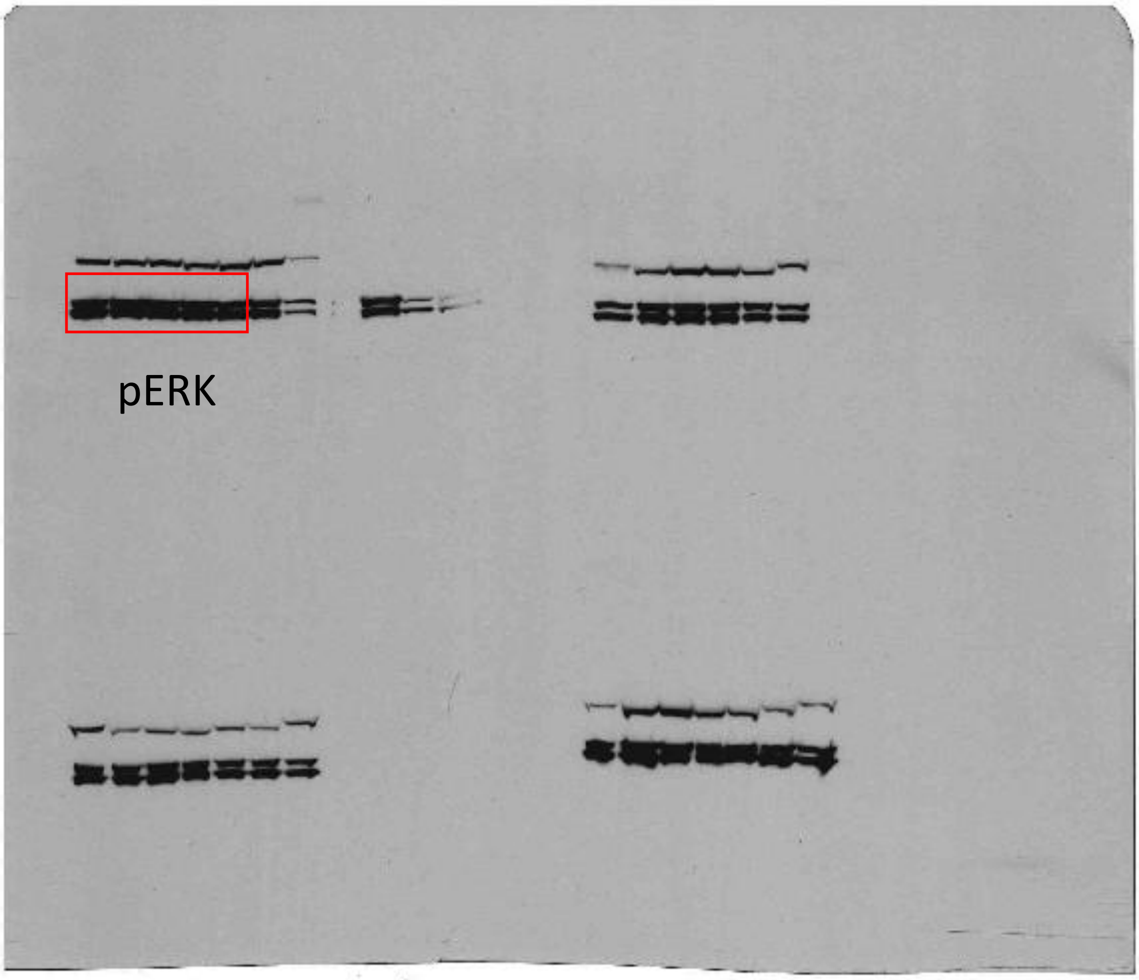

Supplementary Fig. 9e

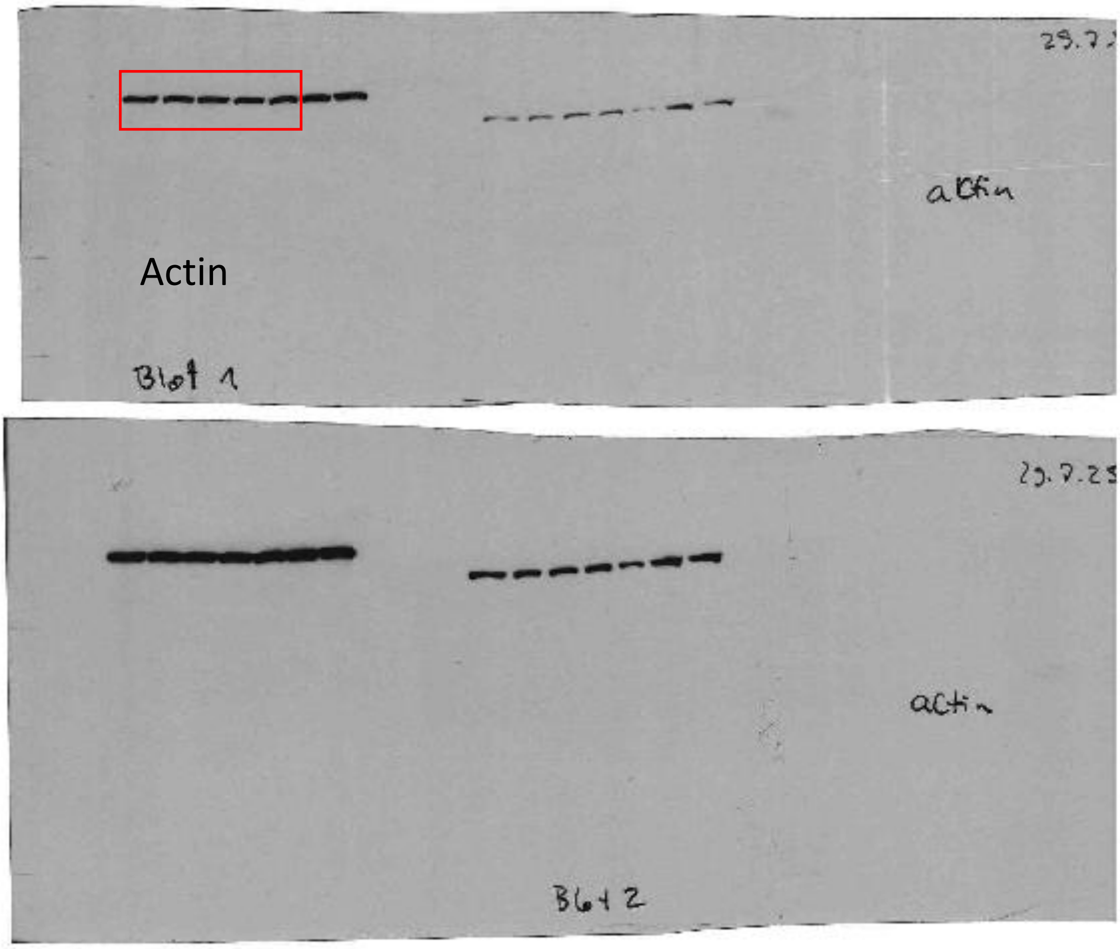

Supplement: Supplementary file 15 — Source Data [file 41467_2025_66800_MOESM15_ESM.zip › Western blot source data_NCOMMS-24-74632B.pdf]
